# Targeting inflammatory and angiogenic responses by small-molecule kinase inhibitors in a 3D macrophage-containing spheroid model of rheumatoid arthritis synovial tissue

**DOI:** 10.1186/s13075-026-03849-z

**Published:** 2026-06-23

**Authors:** Eva M.L. Philippon, Lisanne J. E. van Rooijen, Aldo Jongejan, Gary P. Sims, Jeeho Lee, Lichchavi Rajasinghe, Jan Piet van Hamburg, Conny J. van der Laken, Sander W. Tas

**Affiliations:** 1 Dept. of Rheumatology and Clinical Immunology, Amsterdam Rheumatology & Immunology Center, Amsterdam University Medical Centers, Amsterdam, the Netherlands; 2 Dept. of Experimental Immunology, Amsterdam University Medical Centers, Amsterdam, the Netherlands; 3 Dept of Epidemiology and Data Science, Amsterdam University Medical Centers, Amsterdam, The Netherlands; 4 Immunology Biosciences, Respiratory and Immunology, BioPharmaceuticals R&D, AstraZeneca, Gaithersburg, MD USA

**Keywords:** Rheumatoid arthritis, 3D model, Fibroblasts, Endothelial cells, Macrophages, Drug screening

## Abstract

**Background:**

In rheumatoid arthritis (RA), interactions between macrophages, fibroblast-like synoviocytes (FLS), and endothelial cells (ECs) drive synovial tissue (ST) inflammation and pathological angiogenesis. Preclinical models reflecting this crosstalk are needed to evaluate novel therapies. We developed a human 3D model of RA ST to assess inflammatory mechanisms and responses to small-molecule kinase inhibitors.

**Methods:**

Polarized M1-like or M2-like macrophages were co-cultured with RAFLS and ECs to generate spheroids embedded in a collagen-based scaffold. Spheroids were unstimulated or exposed to pro-inflammatory stimuli, including tumor necrosis factor-α (TNF) or RA synovial fluid (SF). Treatment effects of tofacitinib (JAK1/3 inhibitor) and an IKKβ inhibitor (IKKβi; targeting NF-κB signaling) were evaluated. Readouts included spheroid outgrowth and macrophage distribution quantified by semi-automated image analysis (*n* = 8), cytokine production measured by ELISA/Luminex (*n* = 8), and RNA sequencing to identify differentially expressed genes (*n* = 4).

**Results:**

M1-like macrophages exhibited higher core retention, while M2-like macrophages showed increased outward migration (*p* < 0.05). M1 spheroids demonstrated elevated expression of M1 markers, chemokines, and EC-activating signatures, whereas M2 spheroids upregulated pathways related to matrix remodeling, migration, and immunoregulation (FDR < 0.05). In M1 spheroids, TNF and SF increased macrophage core density compared with unstimulated conditions (*p* < 0.01) and exhibited differential effects on spheroid outgrowth. SF induced RA-associated molecular programs that were broadly suppressed by IKKβi, whereas tofacitinib selectively reduced JAK-dependent signaling (FDR < 0.05). IKKβi significantly decreased TNF-induced mediators (IL-8: *p* < 0.001, M-CSF: *p* < 0.05, SPP1: *p* < 0.001), and tofacitinib primarily inhibited IL-6 production (*p* < 0.01).

**Conclusions:**

This human 3D multicellular in vitro spheroid model of synovial inflammation recapitulates key RA pathological processes and provides a robust platform for mechanistic studies and therapeutic evaluation.

**Supplementary Information:**

The online version contains supplementary material available at https://doi.org/10.1186/s13075-026-03849-z.

## Background

Rheumatoid arthritis (RA) is characterized by disruption of synovial homeostasis, resulting in a highly inflammatory and tissue-destructive microenvironment. Fibroblast-like synoviocytes (FLS) and macrophages become aberrantly activated, driving each other into pathogenic phenotypes that sustain chronic inflammation [1]. FLS secrete cytokines and chemokines to recruit circulating monocytes and promote their differentiation into pro-inflammatory macrophages [2, 3], while activated macrophages enhance FLS proliferation and invasiveness, establishing a self-amplifying inflammatory loop [4]. Endothelial cells (ECs) further contribute by upregulating adhesion molecules and chemokines promoting immune cell recruitment [5], and by producing angiogenic and matrix-degrading mediators that support pathological angiogenesis, and eventually pannus expansion and joint damage [6–8]. Together, these multicellular interactions establish the synovium as the central hub of RA pathology.

The heterogeneity of synovial inflammation contributes to variable treatment responses, with up to 40% patients failing to respond adequately and a subset of around 20% is even classified as difficult-to-treat RA [9, 10]. Dysregulated intracellular signaling networks, including the Nuclear Factor kappa B (NF-κB) and JAnus Kinase and Signal Transducer and Activator of Transcription (JAK/STAT) pathways, are major determinants of pro-inflammatory mediator production and pathogenic cellular reprogramming [11].

Macrophages represent a highly heterogeneous population and are particularly abundant in RA synovial inflammation, with their numbers correlating strongly with disease severity and decreasing in response to effective therapy [12, 13]. They are classically categorized as pro-inflammatory M1 or anti-inflammatory M2 subsets, and therapeutic approaches aimed at promoting M2 polarization are currently under investigation to mitigate inflammation [14, 15]. While this binary M1/M2 classification remains a practical framework for in vitro experimentation, it insufficiently captures the remarkable in vivo plasticity of macrophages. Single-cell RNA-sequencing (scRNA-seq) studies have since refined this view, establishing that etiology-based classification delineates monocyte-derived infiltrating macrophages, largely pro-inflammatory, and tissue-resident macrophages, which comprise both pro-inflammatory and homeostatic states [16–20]. This diversity underscores the need for models that capture macrophage functional states within a physiologically relevant environment.

Three-dimensional (3D) in vitro models have emerged as powerful tools for studying synovial pathology, integrating both the immune and stromal cell compartments, together with extra-cellular matrix (ECM) components to better mimic in vivo interactions [21–23]. We previously incorporated monocyte-derived macrophages into a spheroid-based system consisting of RAFLS and ECs to serve as a 3D multicellular model of key synovial interactions, including inflammatory and angiogenic responses [24, 25]. We demonstrated that stimulation with RA synovial fluid (SF) increases macrophage retention in the spheroid core and enhances pro-inflammatory cytokine expression [26].

Here, we examine how macrophage phenotype influences multicellular behavior and pathological features within this 3D surrogate of RA synovial tissue (ST). Macrophage states are represented using an M1-like and M2-like framework as a simplified approach to model opposing inflammatory states. We then use the SF-stimulated M1-spheroid model to assess the therapeutic effects of small-molecule kinase inhibitors, including the clinically approved JAK1/3 inhibitor tofacitinib and a small-molecule IKKβ inhibitor (IKKβi) targeting canonical NF-κB signaling. Using bulk-RNA sequencing, we characterize SF-driven transcriptional changes and identify pathways modulated by these inhibitors. We provide insights into their mechanism of action within a multicellular, physiologically relevant, in vitro model of RA synovial inflammation.

## Methods

### Patient materials

FLS were isolated from ST biopsies of patients with active RA (RAFLS, 5 donors) [27]. ECs were obtained from human umbilical vein (HUVEC, 5 donors) and monocytes were isolated from healthy donor buffy coats (10 donors) (Sanquin, Amsterdam, NL). SF was aspirated from the knees of RA patients with active disease and clarified by sequential centrifugation. For experiments, SF cocktails were made by pooling synovial fluid of at least 8 RA patients and aliquoted for single use.

### Cell culture

RAFLS, ECs and monocytes were cultured as previously described [26]. Monocytes (3 × 10⁵ cells/well, 24-well plate) were differentiated into macrophages in IMDM medium (Gibco, Thermo Fisher Scientific, San Diego, CA, USA) supplemented with 5% fetal calf serum (FCS; Biowest, Ennigerloh, Germany), 250 µg/ml gentamicin, and 20 ng/ml M-CSF (PeproTech^®^, Gibco) for 6 days, with medium renewal on day 3. Macrophages were polarized for 24 h with IFN-γ (50 ng/ml, M1-like) or IL-10 (50 ng/ml, M2c-like) in culture medium, and harvested on day 7.

### Spectral flow cytometry

Polarized macrophages were stained with fluorochrome-conjugated antibodies from BioLegend (San Diego, CA, USA) against CD163 (1:10, #333607), CD86 (1:100, # 305425), HLA-DR (1:100, # 307643), ICAM-1 (1:400, #353117), TREM2 (1:10, #824805), FRβ (1:100, #391705), MerTK (1:50, #367609) and CD206 (1:10, #321103), diluted in PBA buffer (PBS containing 0.5% BSA, 0.02% NaN₃, and 2 mM EDTA). Apoptic and dead cells were excluded by staining with Fixable Viability Dye eFluor™ 780 (eBioscience, ThermoFisher Scientific). Samples were analyzed on a ID7000 Spectral Cell Analyzer (Sony, Tokyo, Japan).

### 3D spheroid co-culture assay

As previously described [26], CellTracker™-labeled (Invitrogen, Thermo Fisher Scientific) RAFLS, ECs, and macrophages (3.75 × 10⁴, 7.5 × 10⁴, and 3.0 × 10⁴ cells, respectively) were combined in 20% methocel/EGM-2 medium (Lonza, Basel, Switzerland), and seeded into 96 U-bottom plates (Greiner BioOne, Stonehouse, UK) to form spheroids overnight. Spheroids were embedded in 1.5 mg/ml of rat-tail collagen I (BD Biosciences, Oxford, UK) polymerized on the surface of 8-well Ibitreat chamber slides (Ibidi, Martinsried, Germany), and cultured for 40 h in M199 (Gibco) containing 2% FCS, 100 µg/ml penicillin-streptomycin, 50 µg/ml heparin (Gibco), 12.5 µg/ml endothelial cell growth supplement (ECGS) (Corning, NY, USA), and 2 mM L-glutamine (Gibco), with or without stimuli/inhibitors. VEGF/bFGF (10 ng/ml; PeproTech, Cranbury, NJ, USA), TNF (10 ng/ml; PeproTech) or SF (20%) were used as stimuli. Tofacitinib (5.0 µM; Pfizer, NY, USA), IKKβi (2.5 µM; resynthesized by AstraZeneca, Cambridge, UK, following patent procedures: WO2008002246A1), etanercept (100 µg/ml; Pfizer) were added in the stimulation media. The selected concentrations of tofacitinib and IKKβi did not affect RAFLS or HUVEC viability in MTT assays, consistent with previous studies reporting no cytotoxic effects at these concentrations in HUVECs and monocytes/macrophages [28–30].

### Enzyme-linked immunosorbent assay (ELISA) and Luminex analysis

TNF and IL-6 levels in culture supernatants were quantified using commercial antibody pairs (TNF MAb11, eBioscience™, Thermo Fisher Scientific; IL-6, U-CyTech, Utrecht, The Netherlands) following manufacturer’s protocols. IL-8, IL-1β, SPP1, GM-CSF, M-CSF, CXCL9, VEGF were additionally assessed using a customized Luminex multiplex panel (R&D Systems, Bio-Techne, MN, USA) according to the manufacturer’s instructions.

### RNA sequencing and functional analysis

Collagen drops containing spheroids were digested with Liberase™ (0.15 µg/ml, Sigma-Aldrich, St. Louis, MO, USA) and pooled (8 drops per condition) for RNA isolation as previously described [26]. Libraries were generated using the KAPA mRNA Hyperprep kit (Roche, Basel, Switzerland) and sequenced on an NovaSeq 6000 (Illumina, San Diego, CA, USA). Reads were trimmed (Trimmomatic v0.39), aligned to GRCh38 (HISAT2; v2.2.1) and gene-level counts were obtained (HTSeq; v1.99.2) and quality assessed with FastQC (v0.11.9) and dupRadar (v1.0.0). Based on multidimensional scaling and hierarchical clustering, one sample (M1-UNSTIM. donor P60) was identified as an outlier and hence removed from further analysis. Genes with > 2 counts-per-million (CPM) in ≥ 3 samples were retained. Differential expression was performed using the limma-voom framework. Differentially expressed genes (DEGs) were defined as |FC| > 1.5 and *p* < 0.05. These DEGs were used to complete Gene Ontology Biological Process (GO: BP) enrichment analysis with ClusterProfiler. The camera function (limma) was applied for further pathway enrichment using KEGG and Hallmark gene sets (MSigDB). Pathways with FDR < 0.05 (Benjamini-Hochberg correction) were considered significantly enriched. Deconvolution analysis was performed using the 15 RA ST scRNA-seq reference samples from Edalat et al. [31]. Cell type-associated markers were identified using the findMarkers function from the scran package with the Wilcoxon test, and DEGs were assigned based on AUC > 0.7.

### Spheroid imaging and quantitative analysis

Spheroids were fixed with 4% paraformaldehyde (1 h, RT), washed in PBS and imaged by confocal microscopy using a STELLARIS 8 (Leica, Wetzlar, Germany). Two-dimensional (2D) projections generated by CI Convert (v8.2) were analyzed in QuPath (v0.4.0) as described previously [26] to quantify outgrowth and core areas, fluorescence intensity, and cell counts. Data represent mean values from 3 to 7 biological replicates (e.g. individual spheroids).

### Statistics

Statistical analyses were performed using GraphPad Prism (v10.6.0). Statistical significance was defined as *p* < 0.05. Paired *t*-tests were used for two-group comparisons. For > 2 groups, repeated-measures one-way ANOVA followed by Dunnett’s post hoc test was applied. When assumptions of normality and homoscedasticity were not met, nonparametric alternatives were used, namely the Friedman test or the Kruskal-Wallis test.

## Results

### M1-like macrophages remain confined within spheroids, whereas M2-like macrophages disperse throughout the 3D matrix

**Fig. 1:**
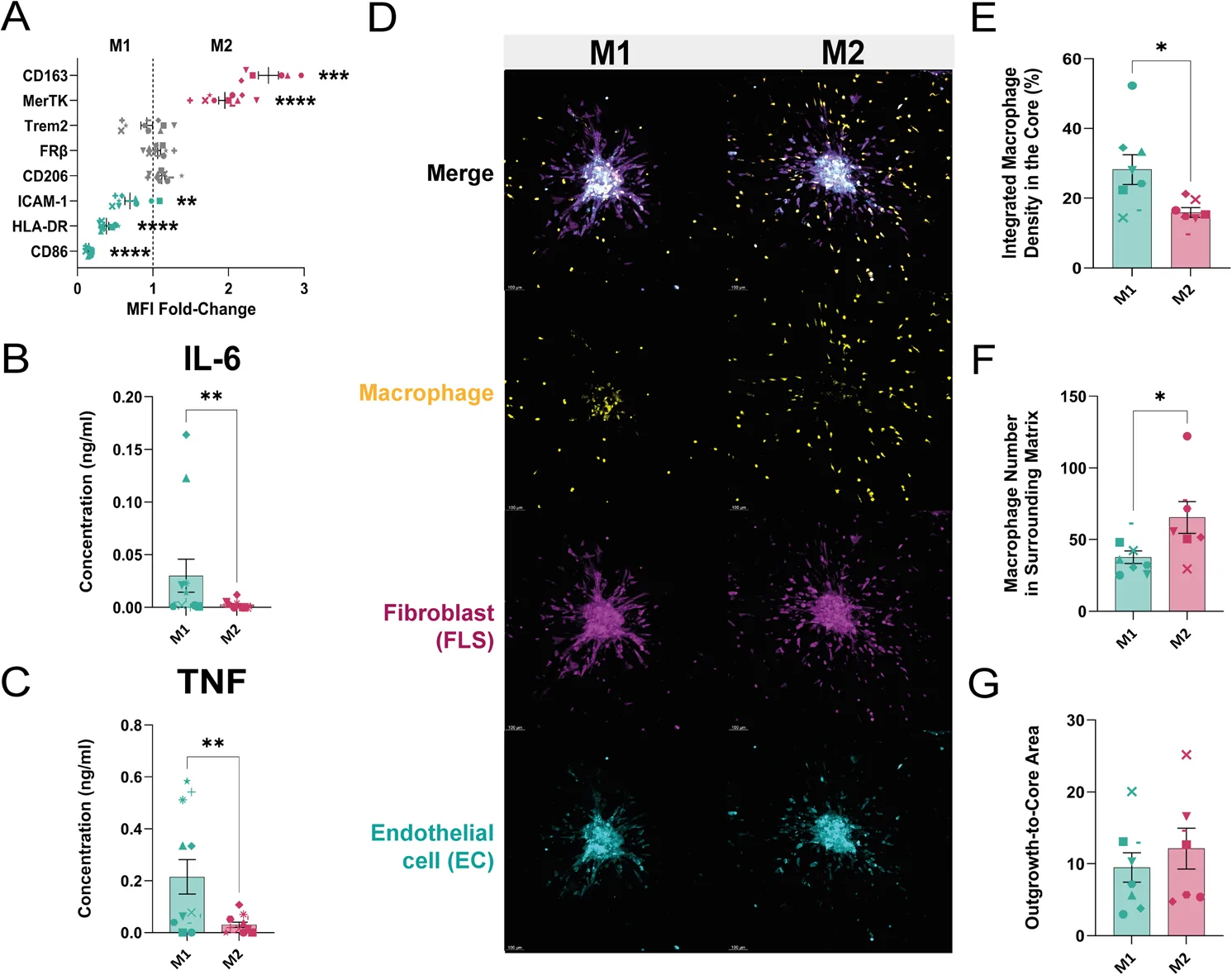
M1-like macrophages were mainly localized to the spheroid core, whereas M2-like macrophages exhibited egress into the surrounding matrix of the 3D model. Macrophages were differentiated from circulating monocytes isolated from fresh peripheral blood of healthy donors in 2D monoculture. **A**: Differential expression of macrophage surface markers in M2-like (> 1) versus M1-like (< 1) subsets after polarization measured by spectral flow cytometry on day 7 (*n* = 10). Values are shown as fold changes in mean fluorescence intensity (MFI). **B**-**C**: IL-6 (**B**) and TNF (**C**) concentrations measured in macrophage culture supernatants determined by ELISA on day 7 (*n* = 12). **D**: Representative confocal pictures of spheroids incorporating M1-like or M2-like macrophages (yellow), RAFLS (magenta) and ECs (cyan) embedded in a collagen-based 3D matrix. Spheroids were fixed and imaged (10X) after 40 h of culture in basal medium. The area covered by the cells that migrated throughout the matrix (outgrowth) was segmented and discriminated from the core area using QuPath (*n* = 7–8). **E**: Percentage of macrophage-specific integrated density ($\:Mean\:Fluorescence\:Intensity\:\times\:Area)\:$quantified in the spheroid core. **F**: Number of macrophages counted in the surrounding matrix. **G**: Outgrowth-to-core area ratio

To better recapitulate the inflammatory synovial interactions of RA, macrophages were integrated into the RAFLS-EC spheroid model. Exploratory differential gene analysis comparing spheroids generated with or without macrophages showed remodeling of the transcriptional landscape toward inflammatory and myeloid-associated programs, highlighting the added value of the tripartite system for modelling the RA synovial microenvironment (Supplementary Material 1). Although in vitro M1-like and M2-like polarized macrophages represent a simplification of the diverse spectrum of macrophages present in vivo, we used them as representative opposite phenotypes. We first examined the expression of key surface markers and cytokine production from both M1-like and M2-like macrophages after polarization in vitro. As expected, M1-like polarized macrophages exhibited elevated surface expression of ICAM-1 (*p* < 0.01), CD86 and HLA-DR (*p* < 0.0001, Fig. 1A), with increased secretion of TNF and IL-6 (*p* < 0.01, Fig. 1B-C). M2-like polarized macrophages expressed higher levels of CD163 and MerTK on their surface (*p* < 0.001, *p* < 0.0001*, respectively*, Fig. 1A). To investigate the effect of these distinct macrophage phenotypes on the 3D model, either M1-like or M2-like macrophages were incorporated in the spheroids together with RAFLS and ECs. While M1-like macrophages remained largely contained within the spheroid core, M2-like macrophages exhibited extensive migration into the surrounding matrix (Fig. 1D). Machine learning-assisted quantification confirmed that M1-like macrophages showed a ~ 2-fold higher integrated density within the spheroid core compared with M2-like macrophages (*p* < 0.05, Fig. 1E), whereas M2-like macrophages harbored a ~ 2-fold increase in cell numbers within the surrounding matrix (*p* < 0.05, Fig. 1F). However, spheroid outgrowth, as marker of FLS migration and angiogenesis, was comparable regardless of the macrophage subset (Fig. 1G, Supplementary Material 2A-B). Moreover, levels of pro-inflammatory mediators (e.g. IL-6, TNF, IL-8, IL-1β, GM-CSF, M-CSF) in culture supernatants did not differ significantly between models (Supplementary Material 2C-H). VEGF levels were too low to be detected in this assay (*data not shown*). These findings demonstrate that both models are characterized by the release of pro-inflammatory cytokines. However, M1-like macrophages exhibit more sustained interactions with RAFLS and ECs within the spheroid core compared with M2-like macrophages.

### M1 spheroids are dominated by chemokine expression and EC-activating programs, while M2 spheroids are defined by matrix-remodeling, immunoregulatory and leukocyte-specific migratory signatures

**Fig. 2:**
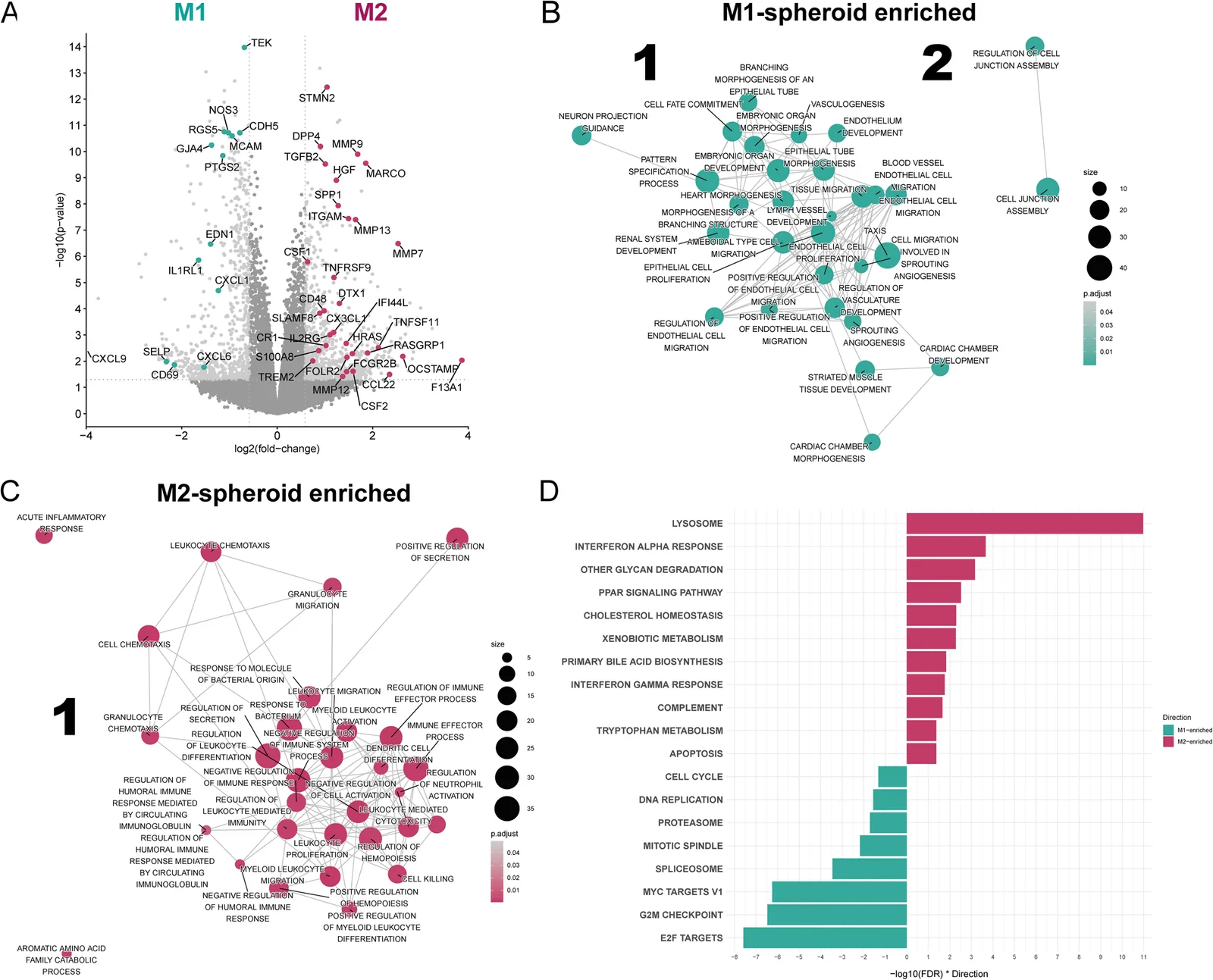
Macrophage polarization shapes the transcriptional landscape of the M1 and M2 spheroids. Bulk-RNA sequencing was performed on spheroids consisting of M1-like (cyan) or M2-like macrophages (magenta), RAFLS and ECs cultured in collagen scaffolds for 40 h under basal conditions (*n* = 4). **A**: Volcano plot showing DEGs (*p* < 0.05, |FC| > 1.5) between M1 and M2 spheroids. RA-relevant genes are highlighted in color and labeled with their HGNC gene name, with CXCL9 (log2(FC) = -12) plotted outside the scale to preserve readability of the remaining data. **B**-**C**: Emapplot depicting Gene Ontology (GO) enrichment analysis (*p*.adjusted < 0.05) of the Biological Process (BP) collection in the M1 spheroids (**B**) or M2 spheroids (**C**). Node connection reflects shared genes between enriched GO terms and node size corresponds to the number of contributing genes. Clusters are identified by numerical labels (1, 2). **D**: Bar plot of enriched KEGG and Hallmark gene sets ranked by significance (FDR < 0.05). The scale represents -log₁₀(FDR) multiplied by directionality (-1: down, + 1: up) with color gradient (cyan: M1-spheroid enriched, magenta: M2-spheroid enriched)

To delineate global transcriptional changes within the multicellular microenvironment driven by macrophage phenotype, bulk-RNA sequencing was performed in the M1- and M2-spheroid models. The DEGs (*p* < 0.05, |FC| > 1.5) are visualized in Fig. 2A, and listed in Supplementary Material 3. Estimated cell-type proportions in the spheroids and cell-type assignments of the top 50 DEGs assigned to each cell type, derived from deconvolution analysis, are provided in Supplementary Material 4A-B. M1 spheroids showed higher expression of genes encoding for chemokines (CXCL1, -6, -9), M1 macrophage-associated markers (PTGS2/COX-2, CD69, IL1RL1/ST2), and genes related to angiogenesis and EC adhesion (SELP, EDN1, GJA4, TEK, MCAM, CDH5, NOS3, RGS5). In contrast, M2 spheroids were enriched for numerous ECM-remodeling proteases (MMP7, -9, -12, -13) and bone/cartilage remodeling markers, such as TNFSF11/RANKL, OCSTAMP, SPP1 and STMN2. Moreover, they expressed several immunomodulatory mediators (TGFB2, DTX1, IL2RG, DPP4), M2-like/resident macrophage-associated markers (ITGAM/CD11b, CCL22, MARCO, TREM2, FOLR2, F13A1), together with the myeloid-associated genes SLAMF8 and S100A8. In addition, increased expression of genes associated with RAS/MAPK signaling (RASGRP1, HRAS), leukocyte trafficking (CSF1/M-CSF, CSF2/GM-CSF, CX3CL1, HGF, TNFRSF9/CD137, CD48), complement activation (CR1) and interferon response (IFI44L) was also observed in M2 spheroids (Fig. 2A). We confirmed the enriched CXCL9 and SPP1 proteins from M1- and M2-spheroid culture supernatants, respectively (Supplementary Material 2I-J). Next, the DEGs were used to perform GO: BP enrichment analysis to identify over-represented biological processes, which were subsequently visualized as networks (Fig. 2B-C). The M1-spheroid model segregated into two main clusters: (1) EC migration/proliferation and vascular development, (2) cell junction assembly (Fig. 2B), while the M2-spheroid model was primarily characterized by a single dominant cluster: (1) leukocyte migration, chemotaxis, and regulation of immune response programs (Fig. 2C). To obtain a pathway-level assessment of coordinated transcriptional changes and account for inter-gene correlations within gene sets, we performed a competitive gene-set enrichment analysis. Analysis of the KEGG and Hallmark gene sets showed preferential activation of proliferative and cell-cycle-associated pathways in M1 spheroids, whereas M2 spheroids displayed stronger induction of immunoregulatory, metabolic and stress-adaptation pathways, including interferon responses, complement activation, cholesterol homeostasis, PPAR signaling, xenobiotic metabolism and apoptosis (Fig. 2D). Consistent with their functional differences, the results indicate that the M1-like and M2-like macrophage subsets elicit distinct molecular signatures in the 3D model of RA ST.

### TNF and SF stimulation enhance macrophage retention within the core in the M1-spheroid model

**Fig. 3:**
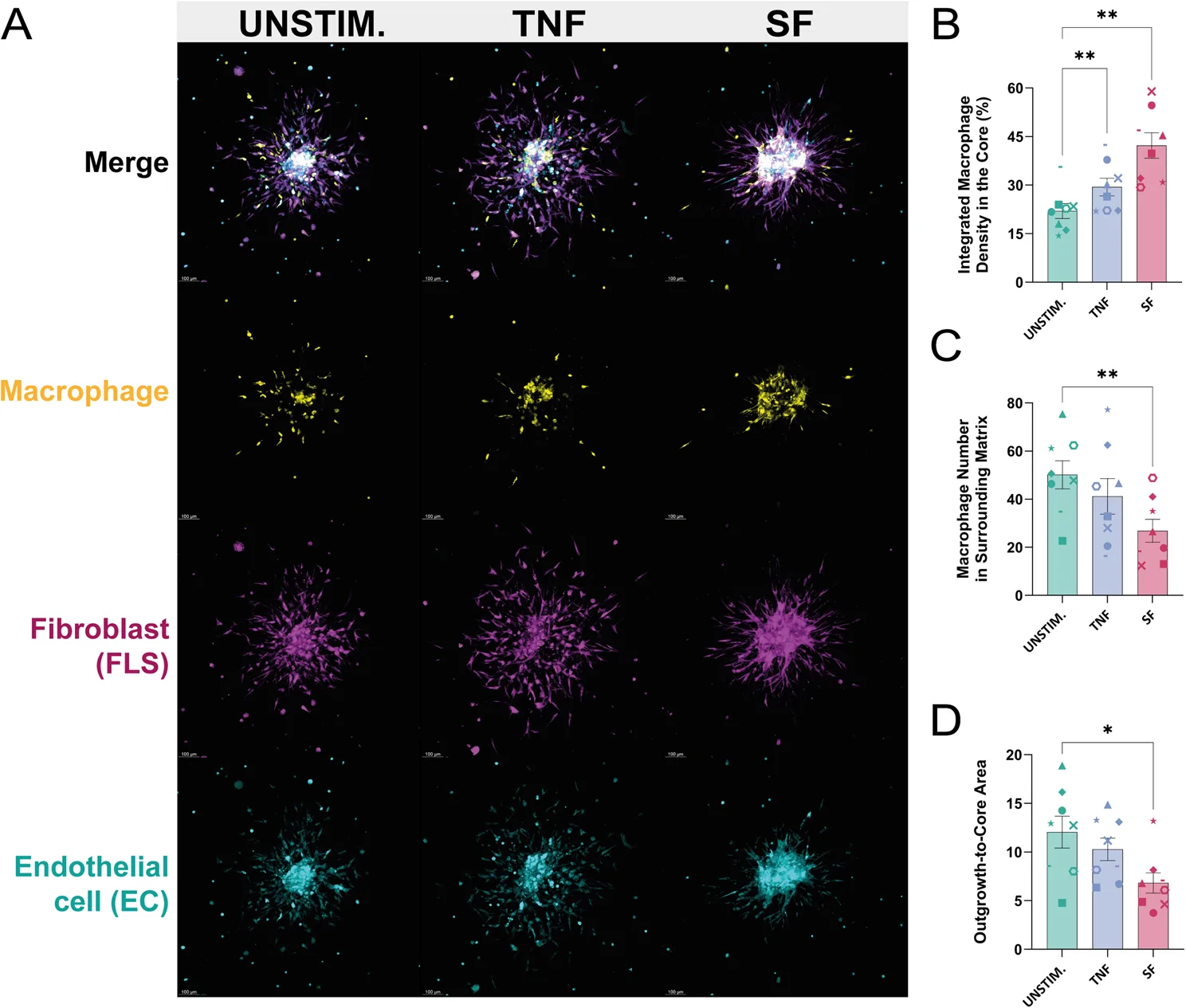
TNF and RA synovial fluid (SF) enhance macrophage retention within the core in the M1-spheroid model. Spheroids containing M1-like macrophages, RAFLS and ECs were embedded in collagen matrices and left unstimulated (UNSTIM.), stimulated with TNF (10 ng/ml) or SF (20%) for 40 h (*n* = 8). **A**: Representative confocal pictures (10X) of the spheroids (M1-like macrophages: yellow, RAFLS: magenta, ECs: cyan) after fixation. The area covered by the cells that migrated throughout the matrix (outgrowth) was segmented and discriminated from the core area using QuPath. **B**: Percentage of macrophage-specific integrated density ($\:Mean\:Fluorescence\:Intensity\:\times\:Area)\:$quantified in the spheroid core. **C**: Number of macrophages counted in the surrounding matrix. **D**: Outgrowth-to-core area ratio

We proceeded with the M1-spheroid model due to its greater macrophage containment. To mimic RA inflammation, two conditions were applied: TNF stimulation, representing a defined cytokine-mediated response, and SF from RA patients, reflecting the multifactorial and in vivo-like synovial microenvironment. Both TNF and SF increased macrophage containment within the core (Fig. 3A). TNF stimulation induced a 1.3-fold increase in macrophage density within the core compared to unstimulated conditions (*p* < 0.01, Fig. 3B). SF stimulation had even more profound effects, boosting macrophage integrated density in the core by almost 2-fold (*p* < 0.01, Fig. 3B) and was reciprocally accompanied by a 2-fold decrease in macrophage numbers in the surrounding matrix (*p* < 0.01, Fig. 3C). Meanwhile, SF exposure reduced spheroid outgrowth (*p* < 0.05, Fig. 3D, Supplementary Material 5A-B), resulting in relatively short, densely organized extensions (Fig. 3A), consistent with prior findings [26]. We previously established this 3D system without macrophages to model RA angiogenesis [25]. To determine whether angiogenesis could still be induced in the macrophage-containing spheroids, stimulation with VEGF/bFGF (GFs) was also applied. Similarly to the prior study [26], GFs strongly promoted spheroid outgrowth, indicative of angiogenic activity, while having no apparent effect on macrophage distribution (Supplementary Material 5A-F). Together, these findings indicate that inflammatory mediators (e.g. TNF and other mediators present in SF) especially impact macrophage behavior in the M1-spheroid model.

### SF activates RA-associated inflammatory, angiogenic, and leukocyte trafficking pathways in the M1-spheroid model

**Fig. 4:**
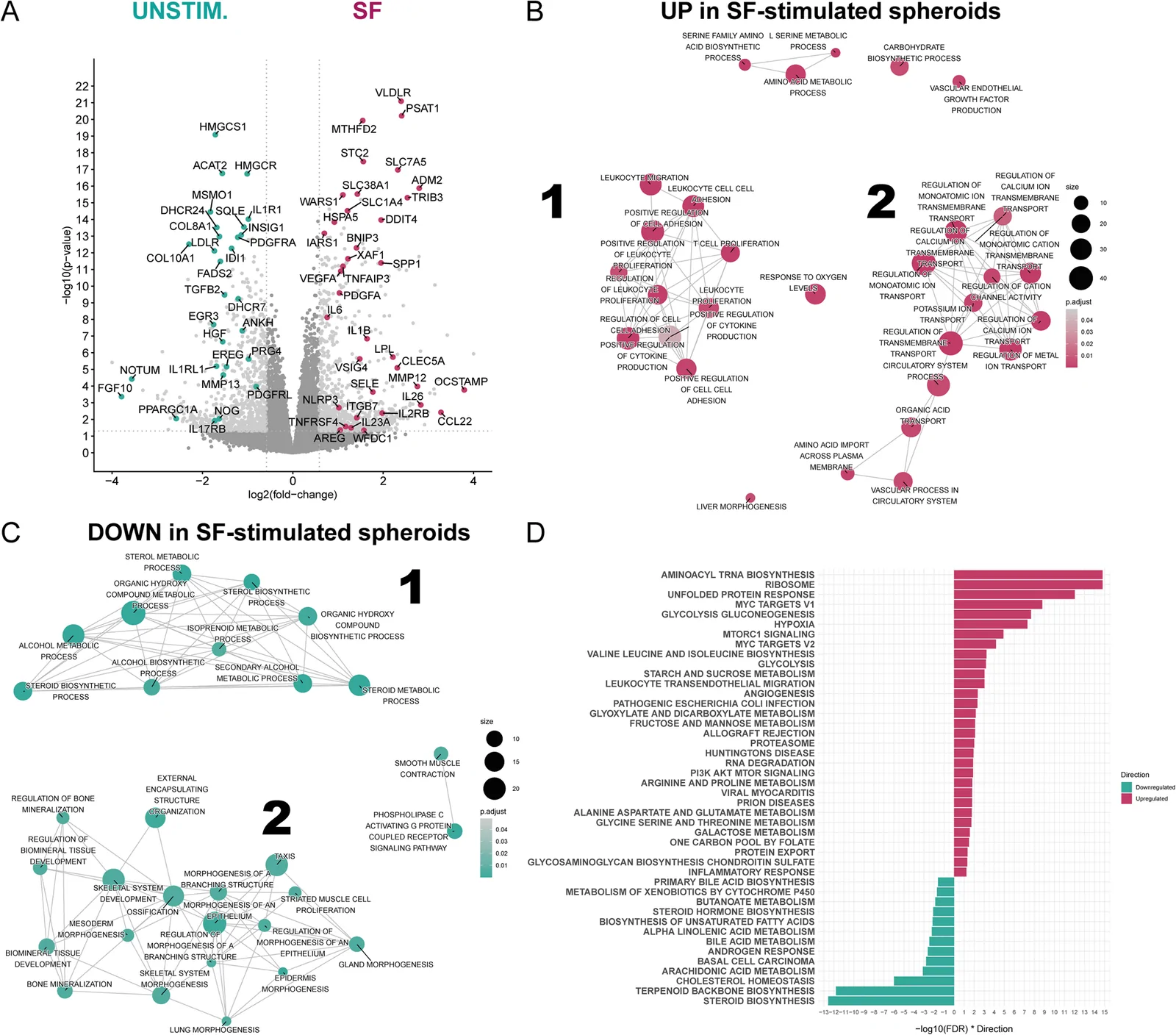
Stimulation with RA synovial fluid (SF) induces RA-like inflammatory and angiogenic transcriptional programs in the M1-spheroid model. Bulk-sequencing was performed in spheroids comprising M1-like macrophages, RAFLS, ECs cultured within collagen drops in basal medium (UNSTIM., cyan) ± supplemented with 20% SF (magenta) for 40 h (*n* = 4). **A**: Volcano plot showing DEGs (*p* < 0.05, |FC| > 1.5) between the two culture conditions. RA-relevant genes are highlighted in color and labeled with their HGNC gene name. **B**-**C**: Emapplot depicting Gene Ontology (GO) enrichment analysis (*p*. adjusted < 0.05) of the Biological Process (BP) collection among genes upregulated in SF-stimulated spheroids (**B**) and downregulated in SF-stimulated spheroids (**C**) relative to unstimulated spheroids. Node connection reflects shared genes between enriched GO terms and node size corresponds to the number of contributing genes. Clusters are identified by numerical labels (1, 2). **D**: Bar plot of enriched KEGG and Hallmark gene sets ranked by significance (FDR < 0.05). The scale represents -log₁₀(FDR) multiplied by directionality (-1: down, + 1: up) with color gradient (cyan: downregulated, magenta: upregulated). The inflammatory response pathway did not meet the significance cutoff (FDR = 0.06) but is presented given its proximity to the threshold and biological relevance

Given that SF showed the strongest effects on spheroid morphology and macrophage distribution, it was used to assess the feasibility of the system as a screening platform. Bulk-RNA sequencing was selected as an initial system-level readout to capture global inflammatory responses emerging from the multicellular microenvironment stimulated by SF. SF exposure led to extensive transcriptional changes, yielding 392 upregulated and 309 downregulated DEGs with |FC| > 2 (Fig. 4A, Supplementary Material 3). Estimated cell-type proportions in the spheroids and cell-type assignments of the top 50 DEGs assigned to each cell type, derived from deconvolution analysis, are provided in Supplementary Material 4A, C. Pro-inflammatory mediators linked to NF-κB and inflammasome-IL-1β signaling were strongly upregulated, including IL1B, IL6, IL23A, IL26, NLRP3, TNFAIP3/A20, CLEC5/MDL1, together with receptors involved in immune regulation (IL2RB, TNFRSF4/OX40, VSIG4). Interestingly, genes previously enriched in M2 spheroids (Fig. 2A) and involved in matrix degradation and bone remodeling (e.g., MMP12, CCL22, OCSTAMP, SPP1) were also found in SF-stimulated M1 spheroids *(*Fig. 4A*)*. In addition, chemokines and adhesion molecules (CXCL11, ITGB7, SELE) were activated, as were pro-angiogenic and fibroblast-activating factors (VEGFA, PDGFA, ADM2, AREG, WFDC1). Metabolic reprogramming was evident through increased expression of stress regulators (DDIT4, TRIB3, STC2, HSPA5, XAF1, BNIP3), amino acid synthesis and transport genes (SLCs, PSAT1, WARS1, EPRS1, IARS1, MTHFD2, VLDLR, LPL), downregulation of cholesterol biosynthesis (HMGCS1, HMGCR, MSMO1, SQLE, INSIG1, DHCR24, DHCR7, IDI1, LDLR), lipid metabolism genes (ACAT2, FADS2) and the peroxisome activator PPARGC1A (Fig. 4A). Growth-related and tissue remodeling genes (FGF10, NOTUM, HGF, TGFB2, EREG, EGR3, PRG4) were suppressed with SF, as were several interleukin receptors (IL17RB, IL1RL1) and matrix-associated genes (MMP13, ANKH, NOG, COL10A1, COL8A1). IL1R1 and PDGFRA/PDGFRL were reversely regulated compared to their upregulated ligand, which may reflect feedback mechanism controlling these axes (Fig. 4A). Mapping of the BPs revealed over-represented pathways among the upregulated and downregulated genes. SF-activated pathways clustered into two main groups: (1) leukocyte proliferation, migration and cell-cell adhesion, and (2) cellular transport and transmembrane trafficking (Fig. 4B), whereas repressed pathways comprised (1) lipid-derived metabolite biosynthesis and metabolism, (2) developmental and morphogenetic processes (Fig. 4C). Subsequent gene-set enrichment analysis of KEGG and Hallmark in SF-stimulated spheroids revealed coordinated activation of inflammatory response, angiogenesis and leukocyte trafficking (transendothelial migration), together with MYC-mTOR-PI3K signaling and hypoxia, hallmarks of proliferative and stress-adaptative states (Fig. 4D). Consistent with higher biosynthetic demand and metabolic reprogramming, upregulated pathways were also associated with enhanced protein synthesis and turnover, as well as increased glycolysis and amino-acid utilization. On the contrary, downregulated pathways were largely related to lipid, sterol, and hormone metabolism, reflecting suppression of lipid-dependent homeostatic programs (Fig. 4D). These results indicate that SF triggers molecular programs linked to RA pathology, encompassing inflammatory and angiogenic responses, EC-macrophage trafficking, matrix-remodeling, and metabolic activation.

### Differential effects of targeting NF-ĸB or JAK/STAT signaling using small-molecule kinase inhibitors in the M1-spheroid model

**Fig. 5:**
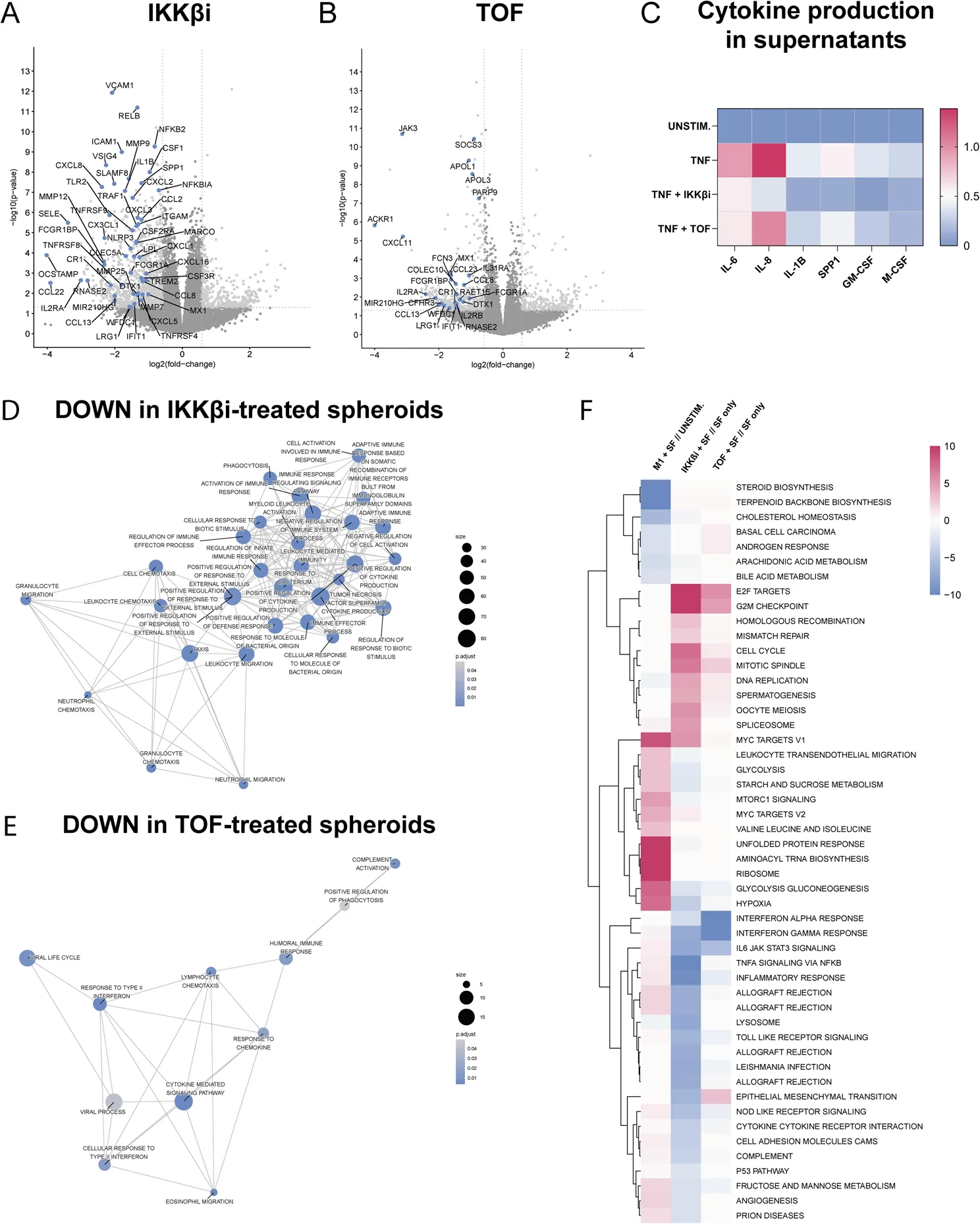
Small-molecule kinase inhibitors of the NF-κB and JAK/STAT pathways suppress key inflammatory markers in the M1-spheroid model. Differential gene expression was assessed by bulk-RNA sequencing between spheroids consisting of M1-like macrophages, RAFLS and ECs embedded in collagen scaffolds and cultured for 40 h in basal medium ± stimuli/inhibitors (*n* = 4). Spheroids were either left unstimulated (UNSTIM.) or stimulated with 20% RA synovial fluid (SF) or 10 ng/mL TNF ± IKKβi (2.5 µM) or tofacitinib (TOF, 5 µM). **A**-**B**: Volcano plot showing DEGs (*p* < 0.05, |FC| > 1.5) between SF-stimulated M1 spheroids treated with IKKβi (**A**) or TOF (**B**) compared to the untreated baseline (SF only). RA-relevant genes are highlighted in color and labeled with their HGNC gene name. **C**: Cytokine concentrations in culture supernatants from TNF-stimulated spheroids treated with IKKβi or TOF, expressed as log2 fold change relative to unstimulated conditions, as measured by ELISA/Luminex (*n* = 7). Raw concentrations are plotted in Supplementary Material 6. **D**-**E**: Emapplot depicting Gene Ontology (GO) enrichment analysis (*p*.adjusted < 0.05) of the Biological Process (BP) collection in the IKKβi-treated (**D**) or tofacitinib-treated (**E**) spheroids (downregulated genes). Node connection reflects shared genes between enriched GO terms and node size corresponds to the number of contributing genes. **F**: Heatmap regrouping the top 50 enriched KEGG and Hallmark gene sets ranked by significance (FDR < 0.05) for each paired comparison: (1) SF versus UNSTIM., (2) IKKβi versus SF only, (3) TOF versus SF only. The scale represents -log₁₀(FDR) multiplied by directionality (-1: down, + 1: up) with color gradient (blue: downregulated, magenta: upregulated)

To investigate the role of key signaling pathways such as NF-κB and JAK/STAT signaling in the M1-spheroid model, we performed bulk-RNA sequencing analysis from the spheroids treated with IKKβi or tofacitinib upon SF stimulation. The DEGs (*p* < 0.05, |FC| > 1.5) based on fold-change values are visualized in Fig. 5A-B, and listed in Supplementary Material 3. IKKβi-treated SF-stimulated M1 spheroids exhibited a broad downregulatory profile, including genes previously induced by SF (IL1B, NLRP3, OCSTAMP, SPP1, TNFRSF4/OX40, VEGFA, AREG, VSIG4, CCL22, MMP12, SELE, ITGAM/CD11b, CLEC5A/MDL-1, WFDC1), alongside newly identified targets such as components of the activation pathways of NF-кB signaling (NFKBIA, TRAF1, RELB, NFKB2), macrophage-associated receptors and regulators (CSF1/M-CSF, CSF2RA, CSF3R, MARCO, SLAMF8, TLR2, TREM2), adhesion molecules (ICAM1, VCAM1), chemokines (CXCL1-3, -5, -8, -16, CCL2, -8, -13, CX3CL1), MMPs (MMP7, -9, -25) and activation-induced immunoregulatory receptors (TNFRSF8, -9, IL2RA) (Fig. 5A). By contrast, tofacitinib elicited a more selective transcriptional response, including a small subset of suppressed genes previously enriched in the SF conditions (OCSTAMP, CXCL11, IL2RB, WFDC1). JAK inhibition primarily suppressed cytokine-driven signaling programs, notably the JAK/STAT cascade (JAK3, SOCS3) and interferon-stimulated gene networks (APOL1, -3, IFIT1, MX1). This translated into dampening of immune effector functions, such as chemokine-driven leukocyte recruitment (CXCL11, CCL8, -13, -23, ACKR1/DARC), cytokine receptor responsiveness (IL2RA, -B, IL31RA), complement activation (CR1, CFHR3, COLEC10, FCN3), NKG2D-mediated stress response (RAET1E) and prostaglandin transport (SLCO2A1) (Fig. 5B). Common downregulated genes across the two treatments comprised the previously mentioned genes (OCSTAMP, CCL8, -13, WFDC1, IFIT1, MX1, IL2RA, CR1), as well as LRG1 (pro-angiogenic factor), FCGR1A, -BP (Fcγ receptor-mediated immune activators), RNASE2 (immune effector protein), MIR210HG (miR-210 host transcript), and DTX1 (NOTCH signaling regulator).

Protein-level expression of key pro-inflammatory cytokines (e.g., IL-6, IL-8, IL-1β, SPP1, GM-CSF, M-CSF) was evaluated to corroborate the transcriptional changes induced by inhibitor treatment. Interpretation of cytokine measurements in SF-stimulated spheroid supernatants is complicated by the fact that SF itself contains large amounts of exogenous cytokines and chemokines, making it difficult to distinguish background from newly produced signals by the cells in the spheroids. To enable a more controlled assessment of compound-mediated effects and demonstrate the cross-platform consistency of the model, soluble inflammatory mediators were quantified in culture supernatants from TNF-stimulated spheroids (Fig. 5C). TNF significantly increased the release of all measured cytokines, whereas IKKβi significantly reduced IL-8, M-CSF, and SPP1 levels, and tofacitinib selectively decreased IL-6 concentrations. Concentration values are plotted in Supplementary Material 6A-H.

GO: BP network analysis identified significant pathways exclusively among downregulated genes (FDR < 0.05), with no significant enrichment observed for upregulated genes. In the IKKβi-treated conditions, the downregulated genes formed a large, highly interconnected module dominated by broad inflammatory and immune processes, including leukocyte activation, chemotaxis and migration (Fig. 5C), while tofacitinib repression was restricted to a narrower set of pathways, primarily complement activation, response to type II interferon, cytokine and chemokine-mediated signaling (Fig. 5D). KEGG and Hallmark gene-set enrichment analysis for both inhibitors were next visualized in a heatmap alongside the results of SF-stimulated spheroids relative to the unstimulated conditions (Fig. 5E). A shared upregulation of cell-cycle pathways was observed across both treatments. IKKβi suppressed SF-induced programs (e.g. inflammatory response, angiogenesis, mTORC1 signaling, hypoxia, glycolysis) and extended its impact to pathways outside the SF response, including TNF signaling via NF-кB, IL6/JAK/STAT3 axis, IFN-α/γ responses, Toll-like receptor signaling, complement activation, cytokine-receptor interaction, cell adhesion and p53 pathway. In contrast, tofacitinib selectively downregulated JAK-dependent immune pathways (IL6/JAK/STAT3 and IFN-α/γ). Despite these strong transcriptional effects, neither inhibitor substantially affected macrophage distribution as assessed by quantitative image analysis although TNFi (etanercept) showed a trend toward reducing macrophage density within the spheroid core (Supplementary Material 7A-B). In the TNF-stimulated model, on the other hand, both tofacitinib and etanercept significantly decreased macrophage core density to baseline levels (Supplementary Material 7C-D). Of note, tofacitinib and IKKβi prevented spheroid outgrowth in the GF-stimulated model (Supplementary Material 7E-G).

## Discussion

Here, we used a previously established spheroid-based model of synovial inflammation incorporating RAFLS, ECs and macrophages, to show that macrophage phenotype defines distinct transcriptional signatures within this 3D model that can be employed to study RA synovial interactions. We also demonstrated that SF stimulation promotes macrophage containment in the M1-spheroid model, accompanied by activation of pathogenic molecular programs. Furthermore, we found that small-molecule kinase inhibitors exhibited drug-specific modulation of SF-induced as well as broader disease-relevant signatures. The model was further validated using other stimuli, including TNF and GFs, highlighting the versality of the system for assessing context-specific drug effects.

Macrophage polarization states were confirmed by the expression of the pan-macrophage subset markers (CD86, CD163) and characterized with additional synovial macrophage markers. Elevated MerTK expression in M2-like macrophages supports their anti-inflammatory phenotype, while increased HLA-DR and ICAM-1 in M1-like macrophages reflect a pro-inflammatory profile, consistent with macrophage populations observed in the RA synovium [16–20]. Macrophage subset-specific functional traits established during polarization were preserved within the 3D ST model. M1 spheroids were enriched in COX-2 and CXC chemokines, characteristic of M1 polarization, while M2 spheroids upregulated MMPs, SPP1, MARCO, CCL22 and F13A1, associated with M2(c) macrophages [32–36]. Pathway analysis revealed heightened apoptotic and phagocytic activity in M2 spheroids, supported by the enrichment of apoptosis, lipid-processing and PPAR-regulated metabolic signatures, consistent with an M2-like phenotype [32, 35, 37, 38]. Nonetheless, activation of IFN-mediated pathways in the M2-spheroid model may imply a shift toward M1-like signaling and/or pathogenic FLS-macrophage crosstalk [32, 39]. In addition, the M2 spheroids were characterized by enhanced osteoclastogenic activity (RANKL, OCSTAMP, (G)M-CSF, CX3CL1) and upregulation of MMP7, -9, -13, involved in cartilage and bone damage, which may indicate tissue-destructive activity [40, 41]. Moreover, several M2-associated genes (CCL22, MMP12, OCSTAMP, SPP1) were also induced in the SF-stimulated M1 spheroids, supporting a potential pro-inflammatory phenotype, in line with the comparable levels of pro-inflammatory soluble factors observed across both models.

Importantly, spatial distribution differed between macrophage subsets. M1-like macrophages showed greater retention within the spheroid core than M2-like macrophages, likely reflecting a combination of chemokine-driven recruitment and adhesion-mediated containment. Higher expression of ICAM-1 on M1-like macrophages may enhance anchoring within the core, while increased local expression of chemokines (CXCL1, -6, -9) in M1 spheroids may further promote immune cell retention [3, 5, 42]. The endothelial compartment also appears to contribute to macrophage containment, as suggested by BP enrichment of EC activation and cell junction pathways. Deconvolution analysis identified ECs as the primary source of SELP, a key mediator of leukocyte-endothelial adhesion [5, 42, 43], which ranked among the most highly upregulated genes in M1 spheroids. Together with upregulation of arterial- and endothelial stabilization-associated genes (ROBO4, CDH5, GJA4) [5, 31, 44], these findings suggest a more mature, structurally cohesive endothelial phenotype within the M1 spheroid core (Supplementary Material 4A-B). However, no phenotypic differences in EC sprouting were observed between the two models. By contrast, M2 macrophage egress was supported by higher expression of MMP9, -12, 13, which have been implicated in endothelial basement membrane and ECM degradation [40]. CX3CL1 and HGF likely further promote macrophage motility [45, 46]. This migratory behavior aligned with the enriched BP pathways in M2 spheroids such as leukocyte proliferation, migration, and chemotaxis. Additional studies will be required to validate the functional contribution of these candidate mechanisms in the observed differences. A similar enrichment profile was recently observed in a synovial-like organoid model containing M2-like macrophages as compared to M1-like macrophages [22]. These observations suggest that macrophage polarization states may influence migration behavior, adhesion properties, or responses to microenvironmental gradients within a 3D environment, features that are not recapitulated in conventional 2D culture systems.

In short, the M1- and M2-spheroid models reflected macrophage-dependent remodeling of the synovial microenvironment. M1 spheroids were defined by macrophage retention, chemokine production and vascular activation, whereas M2 spheroids exhibited migratory, matrix-remodeling, osteoclastogenic and immunoregulatory programs.

Upon further stimulation, the M1-spheroid model exhibited the strongest morphological changes as opposed to its M2 counterpart *(data not shown)*, highlighting the importance of selecting macrophage subsets that are suited to the experimental objective. For example, the M1 model is better suited for maintained stromal cell-macrophage interactions. Various stimuli were used to model distinct pathological processes, including angiogenesis (GFs) and inflammation (TNF, SF). TNF and SF enabled comparison between a defined inflammatory stimulus and a complex patient-derived inflammatory environment, both of which promoted macrophage retention in M1 spheroids, whereas GF stimulation induced spheroid outgrowth. Unlike earlier reports using RAFLS-EC spheroids alone [25], SF did not induce spheroid outgrowth when macrophages were included. Instead, SF reduced the outgrowth-to-core ratio and promoted dense, short-range cell spreading, consistent with another previous study [26]. This may partly reflect increased cell compaction driven by leukocyte-EC adhesion molecules (ITGAM, ITGB2, ICAM1, SELE), chemotactic signaling (CCL2/CCL7/CCL8-CCR1/CCR5), and the anti-angiogenic mediator TNFSF15, all enriched in the tripartite versus FLS/EC-only spheroids (Supplementary Material 1, 4D-E). In the current study, more pronounced effects of SF were observed, which may be due to SF batch variations and/or the difference in macrophage polarization (M-CSF-based differentiation versus GM-CSF only). Of note, this refined protocol has been shown to more closely mimic the phenotype of RA ST macrophages [34]. Altogether, these observations highlight the critical role of myeloid-stromal crosstalk in regulating tissue organization. While HUVECs were used as a robust and widely established endothelial component, the inclusion of microvascular ECs (e.g., HDMECs) may further enhance the disease specificity of the 3D model.

SF exposure activated RA-associated transcriptional programs in M1 spheroids, including inflammatory response, angiogenesis, PI3K/AKT/mTOR cascade, hypoxia and glycolysis. SF stimulation upregulated a gene network centered around IL-1β and IL-6, key mediators sustaining chronic synovial inflammation via the PI3K/AKT/mTOR axis [11], and known to be induced by the NLRP3 inflammasome and IL-26 [47, 48], both also increased. Elevated expression of VEGF and AREG, pro-angiogenic mediators produced by RAFLS and macrophages, which are downstream of IL-1β [8, 49, 50], aligns with this IL-1β-enriched signature. Deconvolution analysis provided additional insight into the cellular contribution to these transcriptional changes, particularly identifying macrophages as the predicted source of the enriched SPP1, NLRP3, and VEGFA signatures. Moreover, the emergence of a mural/perivascular-like transcriptional signature, characterized by enrichment of ABCC9, ACTA2 and PDGFA, suggested acquisition of vascular-support programs within the spheroids upon SF stimulation (Supplementary Material 4A, C). Additionally, upregulation of genes involved in cell-cell adhesion in the RA synovium (SELE, CXCL11, MDL-1), together with enrichment of leukocyte cell-cell adhesion pathways in BP analysis, may contribute to reduced spheroid outgrowth and enhanced macrophage retention [51, 52]. Activation of angiogenesis and leukocyte transendothelial migration pathways in the presence of SF further supported close immune-stromal interactions characteristic of RA. Interestingly, while SF activated similar pathways in the M2-spheroid model, comparative analysis of SF-stimulated M1 and M2 spheroids demonstrated preservation of subset-specific transcriptional signatures observed under basal conditions. Specifically, leukocyte migration and immune effector processes remained enriched in the M2 model, whereas EC activation and cell junction programs persisted in the M1 model (Supplementary Material 8A-I).

Next, the M1-spheroid model was used for therapeutic screening of small-molecule kinase inhibitors upon SF stimulation. NF-κB inhibition by IKKβi resulted in broad transcriptional suppression of macrophage-associated markers (SPP1, VSIG4, M-CSF, MARCO and SLAMF8) [17, 18, 53], chemokines, MMPs and adhesion molecules. In contrast, tofacitinib predominantly affected genes within its canonical target pathway (JAK3, SOCS3). At the pathway level, multiple pathogenic signaling routes were downregulated by IKKβi (TNF-NF-κB, IL6/JAK/STAT3, IFN-α/γ, angiogenesis), whereas tofacitinib selectively inhibited IL-6/JAK/STAT3 and interferon-related pathways. Validation of inhibitory effects at the protein level was hampered by the composition of SF, which intrinsically contains high levels of pre-existing cytokines and chemokines, interfering with the magnitude of drug effects. Consequently, the SF-stimulated system is better suited to capture transcriptional and secretory trends under drug intervention rather than absolute quantitative changes. To address this limitation, complementary stimulation strategies under controlled conditions can be employed (e.g., TNF stimulation), where reduction of key pro-inflammatory factors by the inhibitors further supported the transcriptional findings and cross-platform consistency of the model. Taken together, these results demonstrate inhibitor-specific modulation of inflammatory processes, supporting the relevance of this model for drug screening.

This study aimed to establish the feasibility of the spheroid model to respond to inflammatory stimuli and pharmacological inhibition by providing an integrated transcriptional readout at the whole-tissue level. Detailed mapping of multicellular interactions was beyond the scope of this initial work. Nevertheless, deconvolution analysis using reference scRNA-seq datasets allowed prediction of the likely cellular sources contributing to transcriptional changes within the model. The system therefore provides a versatile platform that can be combined with higher-resolution approaches to dissect discrete cell-cell and cell-matrix interactions. In particular, future integration of scRNA-seq, spatial transcriptomics, and multicolour quantitative co-localization imaging will be important to confirm specific crosstalk in this 3D model system. In addition, coupling this model with perfused organ-on-chip systems could enable investigation of leukocyte trafficking within a more dynamic microenvironment [54].

## Conclusions

This study extends a previously established 3D model of RA ST by investigating the contribution of M1-like and M2-like macrophages within RAFLS-EC spheroids. The two macrophage states imposed distinct phenotypic and transcriptional signatures, underscoring that macrophage polarization is a major determinant of pathological processes and remodeling dynamics in the synovial niche. Therefore, this 3D system offers a valuable platform for dissecting macrophage-driven mechanisms of synovial inflammation. SF-stimulated M1 spheroids displayed RA-associated transcriptional programs and enhanced macrophage retention. Approved and experimental kinase inhibitors differentially attenuated inflammatory pathways in this model, reflecting their mechanism of action. The integration of additional stimuli (e.g. TNF, GFs) further demonstrated the model’s versatility in modeling specific RA pathological processes and its applicability for evaluating mechanism-targeted drug responses.

## Supplementary Information

Supplementary Material 1.

Supplementary Material 2.

Supplementary Material 3.

Supplementary Material 4.

Supplementary Material 5.

Supplementary Material 6.

Supplementary Material 7.

Supplementary Material 8.

Supplementary Material 9. Supplementary Material Legends

## Data Availability

The datasets generated and analyzed during the current study are available in the Gene Expression Omnibus (GEO) repository: https://www.ncbi.nlm.nih.gov/geo/query/acc.cgi?acc=GSE325085.

## Abbreviations

RA: Rheumatoid Arthritis
ST: Synovial Tissue
FLS: Fibroblast-Like Synoviocytes
ECs: Endothelial Cells
TNF: Tumor Necrosis Factor alpha
GFs: Growth Factors
SF: RA Synovial Fluid
UNSTIM: Unstimulated
