# Supplementary material for: Targeting inflammatory and angiogenic responses by small-molecule kinase inhibitors in a 3D macrophage-containing spheroid model of rheumatoid arthritis synovial tissue
Source: Arthritis Res Ther. 2026 Jun 23;28:163. doi: 10.1186/s13075-026-03849-z (PMC13543596; doi:10.1186/s13075-026-03849-z)
Supplement: Supplementary file 1 [file 13075_2026_3849_MOESM4_ESM.pdf]

A

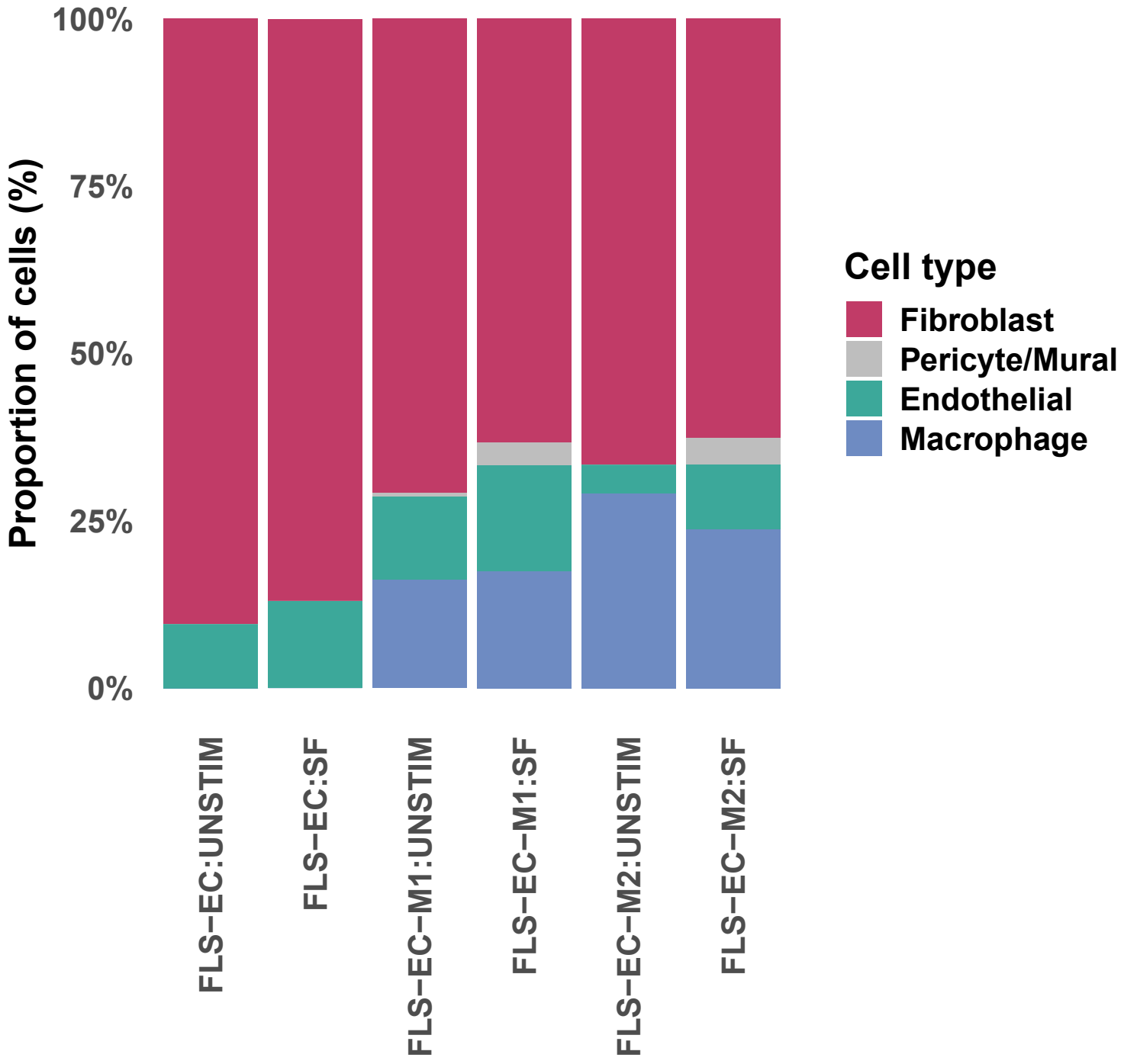
100%
75%
50%
25%
0%
Proportion of cells (%)
FLS−EC:UNSTIM
FLS−EC:SF
FLS−EC−M1:UNSTIM
FLS−EC−M1:SF
FLS−EC−M2:UNSTIM
FLS−EC−M2:SF
Cell type
Fibroblast
Pericyte/Mural
Endothelial
Macrophage

B

Top 50 cell-type assigned genes in unstimulated M2 spheroids (vs M1)

Assigned	Gene	FLS	Mural cells	EC	Macrophages	log2FC
EC	MEOX1	0.20	0.18	0.82	0.18	1.75
EC	PGM5	0.27	0.36	0.73	0.29	1.08
EC	SELP	0.17	0.16	0.84	0.16	−2.32
EC	MMRN1	0.28	0.28	0.72	0.28	−1.49
EC	NOSTRIN	0.17	0.17	0.83	0.17	−1.31
EC	ITGB4	0.17	0.17	0.84	0.16	−1.01
EC	MALL	0.16	0.14	0.87	0.13	−0.94
EC	IL33	0.21	0.20	0.80	0.20	−0.94
EC	MTUS1	0.14	0.24	0.90	0.10	−0.90
EC	PTPRB	0.07	0.07	0.93	0.07	−0.89
EC	ROBO4	0.15	0.15	0.85	0.16	−0.88
EC	GIMAP6	0.20	0.20	0.80	0.63	−0.88
EC	SHE	0.26	0.25	0.74	0.28	−0.84
EC	MMRN2	0.10	0.12	0.90	0.10	−0.82
EC	S1PR1	0.17	0.15	0.83	0.20	−0.81
EC	THSD7A	0.29	0.28	0.72	0.28	−0.81
EC	JAG2	0.28	0.28	0.72	0.28	−0.81
EC	RASIP1	0.18	0.18	0.82	0.18	−0.79
EC	VWF	0.03	0.03	0.97	0.03	−0.79
EC	ESAM	0.08	0.25	0.92	0.08	−0.79
EC	CDH5	0.06	0.07	0.94	0.07	−0.78
EC	CD93	0.03	0.04	0.97	0.10	−0.78
EC	KDR	0.19	0.20	0.81	0.19	−0.77
EC	MYCT1	0.17	0.17	0.83	0.17	−0.77
EC	FLT1	0.08	0.09	0.92	0.11	−0.77
EC	EMCN	0.05	0.06	0.95	0.05	−0.76
EC	GNG11	0.81	0.04	0.99	0.01	−0.76
EC	PREX2	0.14	0.15	0.88	0.12	−0.76
EC	SYNPO	0.26	0.29	0.81	0.19	−0.76
EC	ACVRL1	0.25	0.21	0.82	0.18	−0.76
EC	PDGFB	0.22	0.27	0.78	0.33	−0.75
EC	ICAM2	0.23	0.22	0.77	0.27	−0.75
EC	RHOJ	0.17	0.20	0.86	0.14	−0.73
EC	CLEC14A	0.04	0.04	0.96	0.04	−0.71
EC	TEK	0.21	0.21	0.79	0.21	−0.69
EC	PODXL	0.18	0.18	0.82	0.18	−0.68
EC	ST6GAL1	0.26	0.26	0.74	0.35	−0.68
EC	EFNB2	0.25	0.25	0.76	0.24	−0.68
EC	OCIAD2	0.33	0.28	0.72	0.28	−0.67
EC	GIMAP7	0.12	0.12	0.88	0.22	−0.67
EC	ARHGEF15	0.24	0.25	0.76	0.24	−0.67
EC	FGD5	0.25	0.19	0.77	0.23	−0.66
EC	SPNS2	0.19	0.22	0.81	0.19	−0.66
EC	FLNB	0.19	0.14	0.81	0.19	−0.64
EC	ABLIM1	0.20	0.17	0.86	0.14	−0.64
EC	EGFL7	0.07	0.07	0.94	0.06	−0.63
EC	TIE1	0.14	0.15	0.86	0.14	−0.63
EC	CD200	0.29	0.30	0.73	0.27	−0.62
EC	EPHB4	0.26	0.24	0.77	0.23	−0.62
EC	BCL6B	0.28	0.28	0.72	0.28	−0.61
EC	ECE1	0.12	0.29	0.89	0.11	−0.61
EC	PRKCH	0.17	0.17	0.83	0.19	−0.60
FLS	IGF1	0.71	0.24	0.24	0.29	1.37
FLS	THBS4	0.77	0.23	0.23	0.23	1.27
FLS	PTGES	0.73	0.30	0.28	0.27	0.76
FLS	BHLHE41	0.74	0.29	0.26	0.36	0.76
FLS	MFAP4	0.77	0.29	0.23	0.23	0.70
FLS	GLT8D2	0.82	0.27	0.24	0.18	0.68
FLS	PDPN	0.89	0.11	0.10	0.11	0.63
FLS	COL8A1	0.81	0.25	0.19	0.19	0.62
FLS	FNDC1	0.83	0.18	0.17	0.17	0.60
FLS	SCARA5	0.70	0.31	0.30	0.30	−1.17
FLS	EFEMP1	0.85	0.18	0.18	0.15	−1.13
FLS	PTPRD	0.77	0.25	0.23	0.23	−0.76
FLS	PDGFD	0.74	0.26	0.30	0.26	−0.71
FLS	CRTAC1	0.82	0.22	0.23	0.18	−0.65
FLS	PRELP	0.78	0.33	0.22	0.22	−0.59
Macrophages	F13A1	0.18	0.18	0.18	0.82	3.87
Macrophages	LILRB1	0.20	0.20	0.20	0.80	2.75
Macrophages	MARCO	0.08	0.08	0.08	0.92	1.85
Macrophages	FBP1	0.17	0.17	0.17	0.83	1.55
Macrophages	ITGAM	0.13	0.13	0.13	0.87	1.49
Macrophages	FOLR2	0.21	0.21	0.21	0.79	1.46
Macrophages	FCGR2B	0.14	0.15	0.14	0.86	1.45
Macrophages	SPP1	0.30	0.28	0.35	0.70	1.28
Macrophages	HCST	0.14	0.15	0.14	0.86	1.16
Macrophages	SELPLG	0.25	0.12	0.26	0.75	1.02
Macrophages	PRKCB	0.29	0.03	0.29	0.71	0.92
Macrophages	FGR	0.24	0.25	0.25	0.76	0.92
Macrophages	SLAMF8	0.25	0.25	0.25	0.75	0.89
Macrophages	S100A8	0.15	0.01	0.15	0.85	0.87
Macrophages	IL4I1	0.28	0.27	0.28	0.72	0.85
Macrophages	LY86	0.25	0.25	0.25	0.75	0.79
Macrophages	PLBD1	0.32	0.22	0.19	0.81	0.79
Macrophages	CD4	0.13	0.17	0.12	0.87	0.78
Macrophages	TREM2	0.27	0.27	0.27	0.73	0.75
Macrophages	MS4A4A	0.17	0.17	0.17	0.83	0.74
Macrophages	EPSTI1	0.29	0.22	0.24	0.71	0.72
Macrophages	CTSH	0.16	0.09	0.21	0.84	0.72
Macrophages	MAFB	0.25	0.14	0.09	0.91	0.71
Macrophages	PTAFR	0.19	0.19	0.21	0.81	0.71
Macrophages	ZNF385A	0.28	0.20	0.21	0.72	0.70
Macrophages	TFRC	0.20	0.19	0.18	0.80	0.68
Macrophages	PLEK	0.07	0.08	0.08	0.93	0.68
Macrophages	CD37	0.14	0.14	0.14	0.86	0.66
Macrophages	ITGAX	0.17	0.17	0.17	0.83	0.66
Macrophages	TMEM176B	0.20	0.29	0.14	0.80	0.65
Macrophages	GM2A	0.28	0.25	0.26	0.72	0.65
Macrophages	FTH1	0.05	0.02	0.00	0.95	0.63
Macrophages	KYNU	0.29	0.23	0.24	0.71	0.63
Macrophages	KCNAB2	0.29	0.29	0.29	0.71	0.63
Macrophages	CTSS	0.01	0.01	0.02	0.99	0.63
Macrophages	HCK	0.21	0.21	0.20	0.79	0.62
Macrophages	SIGLEC1	0.24	0.24	0.24	0.76	0.61
Macrophages	MYO1F	0.18	0.18	0.18	0.82	0.60
Macrophages	SPI1	0.05	0.06	0.05	0.95	0.60
Macrophages	PTPN6	0.20	0.21	0.21	0.80	0.59
Macrophages	RASGEF1B	0.11	0.11	0.09	0.89	0.59
Macrophages	EREG	0.29	0.28	0.28	0.71	−0.66
Mural cells	LGALS3	0.70	0.92	0.25	0.75	0.63
Mural cells	CDH6	0.30	0.70	0.30	0.30	−1.61
Mural cells	SLC7A2	0.66	0.83	0.34	0.34	−1.41
Mural cells	RGS5	0.17	0.83	0.18	0.17	−1.11
Mural cells	THBS1	0.38	0.79	0.63	0.37	−0.78
Mural cells	NUDT4	0.61	0.78	0.37	0.39	−0.65

Assigned (AUC > 0.7)
- Fibroblasts
- Pericytes_Mural_cells
- Endothelial_cells
- Macrophages

log2FC
4
2
0
−2
−4

C

Top 50 cell-type assigned genes in SF-stimulated M1 spheroids (vs UNSTIM.)

Assigned	Gene	FLS	Mural cells	EC	Macrophages	log2FC
EC	RAMP3	0.09	0.09	0.92	0.08	2.55
EC	SELE	0.16	0.16	0.84	0.16	1.77
EC	APLNR	0.23	0.22	0.78	0.22	1.57
EC	TESC	0.28	0.30	0.72	0.31	1.10
EC	SLCO2A1	0.10	0.11	0.90	0.10	1.00
EC	CD200	0.29	0.30	0.73	0.27	0.98
EC	PCDH17	0.23	0.23	0.77	0.23	0.84
EC	FAM110D	0.19	0.20	0.81	0.19	0.84
EC	PLVAP	0.04	0.04	0.96	0.04	0.83
EC	FLT1	0.08	0.09	0.92	0.11	0.80
EC	CD34	0.09	0.08	0.92	0.08	0.78
EC	PDGFB	0.22	0.27	0.78	0.33	0.77
EC	HECW2	0.35	0.29	0.72	0.28	0.70
EC	LIMS2	0.22	0.29	0.80	0.20	0.69
EC	VWA1	0.15	0.12	0.91	0.09	0.65
EC	PODXL	0.18	0.18	0.82	0.18	0.64
EC	ETS2	0.09	0.14	0.91	0.71	0.62
EC	MGST2	0.15	0.17	0.85	0.21	0.61
EC	TM4SF18	0.14	0.14	0.86	0.14	0.61
EC	BCAM	0.07	0.72	0.93	0.07	0.60
EC	EBF3	0.36	0.31	0.72	0.28	−0.95
EC	JAM2	0.16	0.12	0.89	0.11	−0.80
EC	ITGA2	0.24	0.24	0.76	0.24	−0.61
EC	MDFIC	0.44	0.15	0.73	0.56	−0.60
FLS	ANK2	0.80	0.33	0.19	0.20	1.19
FLS	CPXM1	0.72	0.34	0.28	0.28	0.64
FLS	CCDC80	0.98	0.14	0.04	0.02	0.63
FLS	COL8A1	0.81	0.25	0.19	0.19	−1.62
FLS	PDGFRA	0.89	0.11	0.11	0.11	−1.22
FLS	PTGES	0.73	0.30	0.28	0.27	−1.18
FLS	ANKH	0.80	0.17	0.20	0.36	−1.12
FLS	ENPP1	0.75	0.29	0.26	0.25	−1.09
FLS	AKR1C1	0.74	0.31	0.36	0.26	−1.05
FLS	PRG4	0.84	0.23	0.17	0.16	−0.98
FLS	GAS1	0.81	0.22	0.20	0.19	−0.95
FLS	LUM	0.99	0.10	0.01	0.01	−0.92
FLS	SNED1	0.80	0.27	0.25	0.20	−0.88
FLS	CYBRD1	0.75	0.28	0.18	0.25	−0.87
FLS	GALNT5	0.75	0.26	0.25	0.25	−0.85
FLS	COLEC12	0.87	0.14	0.13	0.33	−0.82
FLS	PDGFRL	0.86	0.19	0.14	0.14	−0.81
FLS	BICC1	0.84	0.24	0.16	0.16	−0.78
FLS	DPT	0.94	0.07	0.05	0.06	−0.76
FLS	TMEM119	0.71	0.37	0.28	0.29	−0.72
FLS	ZFHX4	0.72	0.30	0.28	0.28	−0.64
FLS	BHLHE41	0.74	0.29	0.26	0.36	−0.64
FLS	CYP1B1	0.83	0.12	0.12	0.17	−0.60
Macrophages	LILRB1	0.20	0.20	0.20	0.80	2.92
Macrophages	SPP1	0.30	0.28	0.35	0.70	1.95
Macrophages	ALOX5AP	0.25	0.01	0.26	0.75	1.70
Macrophages	VSIG4	0.12	0.12	0.12	0.88	1.48
Macrophages	HCST	0.14	0.15	0.14	0.86	1.42
Macrophages	IL1RN	0.30	0.30	0.30	0.70	1.39
Macrophages	FBP1	0.17	0.17	0.17	0.83	1.16
Macrophages	VEGFA	0.34	0.30	0.21	0.79	1.06
Macrophages	ITGAM	0.13	0.13	0.13	0.87	1.02
Macrophages	NLRP3	0.29	0.29	0.29	0.71	1.02
Macrophages	RGCC	0.32	0.10	0.19	0.81	0.97
Macrophages	PLEK	0.07	0.08	0.08	0.93	0.95
Macrophages	CSTA	0.30	0.29	0.29	0.70	0.79
Macrophages	CORO1A	0.24	0.04	0.24	0.76	0.78
Macrophages	ATF5	0.26	0.28	0.27	0.74	0.75
Macrophages	SLC16A3	0.29	0.18	0.20	0.71	0.75
Macrophages	IL4I1	0.28	0.27	0.28	0.72	0.74
Macrophages	ARHGAP4	0.28	0.28	0.32	0.72	0.71
Macrophages	PGK1	0.26	0.22	0.29	0.74	0.70
Macrophages	ADAP2	0.17	0.24	0.17	0.83	0.59
Macrophages	EREG	0.29	0.28	0.28	0.71	−1.47
Macrophages	RGS2	0.19	0.13	0.13	0.81	−1.44
Macrophages	INSIG1	0.23	0.18	0.26	0.77	−1.14
Macrophages	CXCL2	0.24	0.21	0.20	0.76	−1.12
Macrophages	NR4A2	0.18	0.17	0.14	0.82	−0.89
Macrophages	TMEM176B	0.20	0.29	0.14	0.80	−0.80
Macrophages	DSC2	0.24	0.24	0.24	0.76	−0.75
Macrophages	EVI2A	0.32	0.23	0.22	0.78	−0.72
Macrophages	HMOX1	0.19	0.13	0.18	0.81	−0.71
Macrophages	KCTD12	0.16	0.10	0.24	0.84	−0.69
Macrophages	SPINT2	0.25	0.12	0.25	0.75	−0.69
Macrophages	MS4A7	0.09	0.10	0.09	0.91	−0.68
Macrophages	MBP	0.24	0.22	0.21	0.76	−0.63
Macrophages	GAS7	0.36	0.22	0.25	0.75	−0.62
Mural cells	ABCC9	0.16	0.84	0.16	0.16	1.32
Mural cells	PDGFA	0.65	0.80	0.60	0.35	1.03
Mural cells	HSPA5	0.64	0.80	0.33	0.36	0.92
Mural cells	ACTA2	0.63	0.92	0.37	0.37	0.91
Mural cells	TAGLN	0.71	0.98	0.04	0.29	0.85
Mural cells	HES4	0.59	0.80	0.24	0.41	0.80
Mural cells	GAPDH	0.37	0.87	0.38	0.63	0.60
Mural cells	CDH6	0.30	0.70	0.30	0.30	−1.22
Mural cells	PDE5A	0.57	0.84	0.16	0.43	−1.00
Mural cells	SLC7A2	0.66	0.83	0.34	0.34	−0.76
Mural cells	EPHX1	0.63	0.82	0.38	0.37	−0.71
Mural cells	ZFP36L1	0.64	0.88	0.36	0.64	−0.62
Mural cells	CCDC102B	0.23	0.78	0.22	0.22	−0.60

Assigned (AUC > 0.7)
- Fibroblasts
- Pericytes_Mural_cells
- Endothelial_cells
- Macrophages

log2FC: 4, 2, 0, −2, −4

D

Top 50 cell-type assigned genes in unstimulated M1 spheroids (vs FLS-EC)

Group	Gene	FLS	Mural cells	EC	Macrophages	log2FC
EC	IL33	0.21	0.20	0.80	0.20	1.22
EC	CCDC69	0.20	0.17	0.80	0.28	1.10
EC	MMRN1	0.28	0.28	0.72	0.28	1.10
EC	MALL	0.16	0.14	0.87	0.13	1.05
EC	MCTP1	0.09	0.09	0.91	0.72	1.00
EC	ITGB4	0.17	0.17	0.84	0.16	0.84
EC	THSD7A	0.29	0.28	0.72	0.28	0.71
EC	GIMAP6	0.20	0.20	0.80	0.63	0.66
EC	PDGFB	0.22	0.27	0.78	0.33	0.64
EC	MTUS1	0.14	0.24	0.90	0.10	0.60
EC	ITM2A	0.14	0.10	0.90	0.10	−2.43
EC	YWHAE	0.28	0.27	0.72	0.34	−1.09
EC	APLNR	0.23	0.22	0.78	0.22	−0.85
EC	CD34	0.09	0.08	0.92	0.08	−0.63
FLS	ITGB8	0.72	0.27	0.27	0.28	1.31
FLS	BHLHE41	0.74	0.29	0.26	0.36	1.25
FLS	EFEMP1	0.85	0.18	0.18	0.15	0.73
FLS	CYP1B1	0.83	0.12	0.12	0.17	0.68
FLS	PLA2G2A	0.84	0.17	0.14	0.16	−0.97
FLS	GLT8D2	0.82	0.27	0.24	0.18	−0.83
FLS	PTN	0.77	0.33	0.23	0.23	−0.82
FLS	COL8A1	0.81	0.25	0.19	0.19	−0.75
FLS	MFAP4	0.77	0.29	0.23	0.23	−0.74
FLS	FBLN1	0.81	0.27	0.19	0.19	−0.71
FLS	ANGPTL1	0.82	0.66	0.18	0.18	−0.67
FLS	SNED1	0.80	0.27	0.25	0.20	−0.66
FLS	MXRA8	0.95	0.20	0.05	0.05	−0.64
FLS	FNDC1	0.83	0.18	0.17	0.17	−0.64
FLS	PDGFRL	0.86	0.19	0.14	0.14	−0.64
FLS	PTGES	0.73	0.30	0.28	0.27	−0.62
FLS	NBL1	0.94	0.14	0.12	0.06	−0.62
FLS	LRRC15	0.80	0.21	0.20	0.20	−0.62
Macrophages	CYBB	0.03	0.03	0.03	0.97	12.83
Macrophages	CD84	0.13	0.13	0.13	0.87	12.70
Macrophages	C1QC	0.15	0.15	0.16	0.85	12.45
Macrophages	LYZ	0.01	0.02	0.01	0.99	11.90
Macrophages	MS4A7	0.09	0.10	0.09	0.91	11.72
Macrophages	TYROBP	0.01	0.01	0.01	0.99	11.27
Macrophages	CD53	0.07	0.07	0.07	0.93	11.26
Macrophages	PLEK	0.07	0.08	0.08	0.93	11.07
Macrophages	SYK	0.16	0.16	0.17	0.84	10.95
Macrophages	SPI1	0.05	0.06	0.05	0.95	10.73
Macrophages	VSIG4	0.12	0.12	0.12	0.88	10.70
Macrophages	IRF8	0.23	0.01	0.22	0.77	10.62
Macrophages	S100A9	0.04	0.06	0.04	0.96	10.57
Macrophages	FPR3	0.11	0.11	0.11	0.89	10.55
Macrophages	CD163	0.08	0.09	0.09	0.92	10.42
Macrophages	ARHGAP30	0.21	0.21	0.21	0.79	10.31
Macrophages	MS4A4A	0.17	0.17	0.17	0.83	10.30
Macrophages	TMEM176B	0.20	0.29	0.14	0.80	10.23
Macrophages	SLA	0.29	0.20	0.29	0.71	9.96
Macrophages	MYO1G	0.20	0.20	0.20	0.80	9.67
Macrophages	SIGLEC1	0.24	0.24	0.24	0.76	9.65
Macrophages	MARCO	0.08	0.08	0.08	0.92	9.61
Macrophages	PARVG	0.27	0.27	0.29	0.73	9.41
Macrophages	ITGAM	0.13	0.13	0.13	0.87	9.31
Macrophages	CSF3R	0.27	0.06	0.27	0.73	9.17
Macrophages	IKZF1	0.27	0.15	0.27	0.73	9.11
Macrophages	TREM2	0.27	0.27	0.27	0.73	9.03
Macrophages	PTPRC	0.03	0.03	0.03	0.97	8.66
Macrophages	NCKAP1L	0.13	0.13	0.13	0.87	8.57
Macrophages	PIK3AP1	0.22	0.22	0.22	0.78	8.17
Macrophages	DOK2	0.17	0.17	0.17	0.83	8.14
Macrophages	CD4	0.13	0.17	0.12	0.87	8.10
Macrophages	LY86	0.25	0.25	0.25	0.75	8.05
Macrophages	MNDA	0.12	0.14	0.13	0.88	7.87
Macrophages	NCF4	0.24	0.24	0.25	0.76	7.80
Macrophages	CYTH4	0.22	0.22	0.22	0.78	7.80
Macrophages	HCK	0.21	0.21	0.20	0.79	7.78
Macrophages	CLEC7A	0.10	0.11	0.11	0.90	7.77
Macrophages	TAGAP	0.27	0.27	0.29	0.73	7.75
Macrophages	SLC11A1	0.14	0.15	0.14	0.86	7.66
Macrophages	NCF1	0.12	0.13	0.13	0.88	7.65
Macrophages	ADAP2	0.17	0.24	0.17	0.83	7.52
Macrophages	MRC1	0.16	0.16	0.16	0.84	7.52
Macrophages	SLCO2B1	0.20	0.20	0.21	0.80	7.40
Macrophages	MSR1	0.12	0.12	0.12	0.88	7.37
Macrophages	CPVL	0.12	0.12	0.17	0.88	7.35
Macrophages	FGR	0.24	0.25	0.25	0.76	7.19
Macrophages	PTAFR	0.19	0.19	0.21	0.81	7.08
Macrophages	PTPN6	0.20	0.21	0.21	0.80	7.07
Macrophages	C5AR1	0.07	0.08	0.07	0.93	6.82
Mural cells	SLC7A2	0.66	0.83	0.34	0.34	1.26
Mural cells	CDH6	0.30	0.70	0.30	0.30	1.06
Mural cells	LGALS3	0.70	0.92	0.25	0.75	0.97
Mural cells	RGS5	0.17	0.83	0.18	0.17	0.81
Mural cells	KIAA0040	0.28	0.72	0.30	0.28	0.72
Mural cells	CPE	0.59	0.80	0.20	0.41	−0.71
Mural cells	LDHA	0.72	0.87	0.28	0.36	−0.70

Assigned (AUC > 0.7)
- Fibroblasts
- Pericytes_Mural_cells
- Endothelial_cells
- Macrophages

log2FC: 20, 10, 0, −10, −20

E

Top 50 cell-type assigned genes in SF-stimulated M1 spheroids (vs FLS-EC)

Gene	Assigned	FLS	Mural cells	EC	Macrophages	log2FC
SELE	EC	0.16	0.16	0.84	0.16	1.86
TESC	EC	0.28	0.30	0.72	0.31	1.58
MMRN1	EC	0.28	0.28	0.72	0.28	1.43
CCDC69	EC	0.20	0.17	0.80	0.28	1.13
MCTP1	EC	0.09	0.09	0.91	0.72	1.09
MTUS1	EC	0.14	0.24	0.90	0.10	0.92
ST6GAL1	EC	0.26	0.26	0.74	0.35	0.70
THSD7A	EC	0.29	0.28	0.72	0.28	0.70
CRIM1	EC	0.09	0.74	0.92	0.08	0.68
PCDH17	EC	0.23	0.23	0.77	0.23	0.67
GIMAP6	EC	0.20	0.20	0.80	0.63	0.67
PREX2	EC	0.14	0.15	0.88	0.12	0.66
ENTPD1	EC	0.13	0.29	0.87	0.70	0.66
PDGFB	EC	0.22	0.27	0.78	0.33	0.62
CLIC2	EC	0.24	0.19	0.76	0.33	0.61
ITGB4	EC	0.17	0.17	0.84	0.16	0.61
OLFM1	EC	0.26	0.28	0.76	0.24	−0.70
EBF3	EC	0.36	0.31	0.72	0.28	−0.69
ITGB8	FLS	0.72	0.27	0.27	0.28	3.01
BHLHE41	FLS	0.74	0.29	0.26	0.36	2.00
EFEMP1	FLS	0.85	0.18	0.18	0.15	1.12
ISLR	FLS	0.95	0.09	0.05	0.05	−1.85
MFAP4	FLS	0.77	0.29	0.23	0.23	−1.21
PRG4	FLS	0.84	0.23	0.17	0.16	−1.18
COMP	FLS	0.85	0.18	0.15	0.15	−1.05
MXRA5	FLS	0.91	0.15	0.11	0.09	−0.86
SSC5D	FLS	0.84	0.20	0.15	0.16	−0.86
LRRC15	FLS	0.80	0.21	0.20	0.20	−0.80
MXRA8	FLS	0.95	0.20	0.05	0.05	−0.79
ANKH	FLS	0.80	0.17	0.20	0.36	−0.79
CRLF1	FLS	0.74	0.29	0.26	0.26	−0.77
TMEM119	FLS	0.71	0.37	0.28	0.29	−0.76
ENPP1	FLS	0.75	0.29	0.26	0.25	−0.75
DPT	FLS	0.94	0.07	0.05	0.06	−0.71
FNDC1	FLS	0.83	0.18	0.17	0.17	−0.70
FBLN1	FLS	0.81	0.27	0.19	0.19	−0.69
NBL1	FLS	0.94	0.14	0.12	0.06	−0.69
SNED1	FLS	0.80	0.27	0.25	0.20	−0.68
CHI3L2	FLS	0.78	0.27	0.22	0.22	−0.68
GLT8D2	FLS	0.82	0.27	0.24	0.18	−0.67
C1S	FLS	0.98	0.82	0.01	0.02	−0.66
THBS2	FLS	0.90	0.17	0.10	0.10	−0.66
AKR1C1	FLS	0.74	0.31	0.36	0.26	−0.66
GFPT2	FLS	0.82	0.20	0.18	0.18	−0.65
MEDAG	FLS	0.79	0.30	0.24	0.21	−0.62
LTBP1	FLS	0.89	0.72	0.18	0.11	−0.61
SMOC1	FLS	0.76	0.24	0.24	0.24	−0.60
MMP14	FLS	0.74	0.26	0.22	0.26	−0.59
PDGFRA	FLS	0.89	0.11	0.11	0.11	−0.59
CD84	Macrophages	0.13	0.13	0.13	0.87	12.93
CD53	Macrophages	0.07	0.07	0.07	0.93	11.57
PTPRC	Macrophages	0.03	0.03	0.03	0.97	11.45
TYROBP	Macrophages	0.01	0.01	0.01	0.99	11.35
MS4A7	Macrophages	0.09	0.10	0.09	0.91	11.07
CYBB	Macrophages	0.03	0.03	0.03	0.97	10.97
C1QC	Macrophages	0.15	0.15	0.16	0.85	10.84
S100A9	Macrophages	0.04	0.06	0.04	0.96	10.75
IRF8	Macrophages	0.23	0.01	0.22	0.77	10.68
VSIG4	Macrophages	0.12	0.12	0.12	0.88	10.63
ITGAM	Macrophages	0.13	0.13	0.13	0.87	10.37
SLA	Macrophages	0.29	0.20	0.29	0.71	10.24
SIGLEC1	Macrophages	0.24	0.24	0.24	0.76	10.09
CD163	Macrophages	0.08	0.09	0.09	0.92	9.94
PLEK	Macrophages	0.07	0.08	0.08	0.93	9.75
MARCO	Macrophages	0.08	0.08	0.08	0.92	9.71
TREM2	Macrophages	0.27	0.27	0.27	0.73	9.61
CLEC7A	Macrophages	0.10	0.11	0.11	0.90	9.41
MNDA	Macrophages	0.12	0.14	0.13	0.88	9.39
SPI1	Macrophages	0.05	0.06	0.05	0.95	9.37
IL1RN	Macrophages	0.30	0.30	0.30	0.70	9.23
PARVG	Macrophages	0.27	0.27	0.29	0.73	9.16
PIK3AP1	Macrophages	0.22	0.22	0.22	0.78	9.11
SYK	Macrophages	0.16	0.16	0.17	0.84	9.04
PTAFR	Macrophages	0.19	0.19	0.21	0.81	8.75
FPR3	Macrophages	0.11	0.11	0.11	0.89	8.70
SLCO2B1	Macrophages	0.20	0.20	0.21	0.80	8.70
CSF3R	Macrophages	0.27	0.06	0.27	0.73	8.69
FGR	Macrophages	0.24	0.25	0.25	0.76	8.56
DOK2	Macrophages	0.17	0.17	0.17	0.83	8.53
MSR1	Macrophages	0.12	0.12	0.12	0.88	8.36
FCGR1A	Macrophages	0.22	0.23	0.22	0.78	8.35
S100A8	Macrophages	0.15	0.01	0.15	0.85	8.29
LY86	Macrophages	0.25	0.25	0.25	0.75	8.28
SLC11A1	Macrophages	0.14	0.15	0.14	0.86	8.14
NCKAP1L	Macrophages	0.13	0.13	0.13	0.87	8.11
MRC1	Macrophages	0.16	0.16	0.16	0.84	7.94
TMEM176B	Macrophages	0.20	0.29	0.14	0.80	7.89
MS4A4A	Macrophages	0.17	0.17	0.17	0.83	7.86
IKZF1	Macrophages	0.27	0.15	0.27	0.73	7.62
NCF4	Macrophages	0.24	0.24	0.25	0.76	7.62
TAGAP	Macrophages	0.27	0.27	0.29	0.73	7.50
SORL1	Macrophages	0.22	0.24	0.23	0.78	7.42
ARHGAP30	Macrophages	0.21	0.21	0.21	0.79	7.37
MS4A6A	Macrophages	0.04	0.04	0.04	0.96	7.22
C3AR1	Macrophages	0.16	0.16	0.16	0.84	7.15
CCR1	Macrophages	0.17	0.17	0.17	0.83	6.98
PTPN6	Macrophages	0.20	0.21	0.21	0.80	6.93
SPINT2	Macrophages	0.25	0.12	0.25	0.75	6.75
IL18	Macrophages	0.28	0.28	0.28	0.72	6.74
CXCR4	Macrophages	0.16	0.17	0.17	0.84	−0.67
VEGFA	Macrophages	0.34	0.30	0.21	0.79	−0.62
SLC7A2	Mural cells	0.66	0.83	0.34	0.34	1.31
LGALS3	Mural cells	0.70	0.92	0.25	0.75	1.00
RGS5	Mural cells	0.17	0.83	0.18	0.17	0.71
KIAA0040	Mural cells	0.28	0.72	0.30	0.28	0.67
THBS1	Mural cells	0.38	0.79	0.63	0.37	0.59
TMEM14C	Mural cells	0.67	0.83	0.63	0.33	−2.40
AOC3	Mural cells	0.28	0.73	0.58	0.42	−0.98
MT−RNR1	Mural cells	0.40	0.83	0.65	0.60	−0.74
CPE	Mural cells	0.59	0.80	0.20	0.41	−0.63

Assigned (AUC > 0.7)
- Fibroblasts
- Pericytes_Mural_cells
- Endothelial_cells
- Macrophages

log2FC
20
10
0
−10
−20
