# Supplementary material for: Targeting inflammatory and angiogenic responses by small-molecule kinase inhibitors in a 3D macrophage-containing spheroid model of rheumatoid arthritis synovial tissue
Source: Arthritis Res Ther. 2026 Jun 23;28:163. doi: 10.1186/s13075-026-03849-z (PMC13543596; doi:10.1186/s13075-026-03849-z)

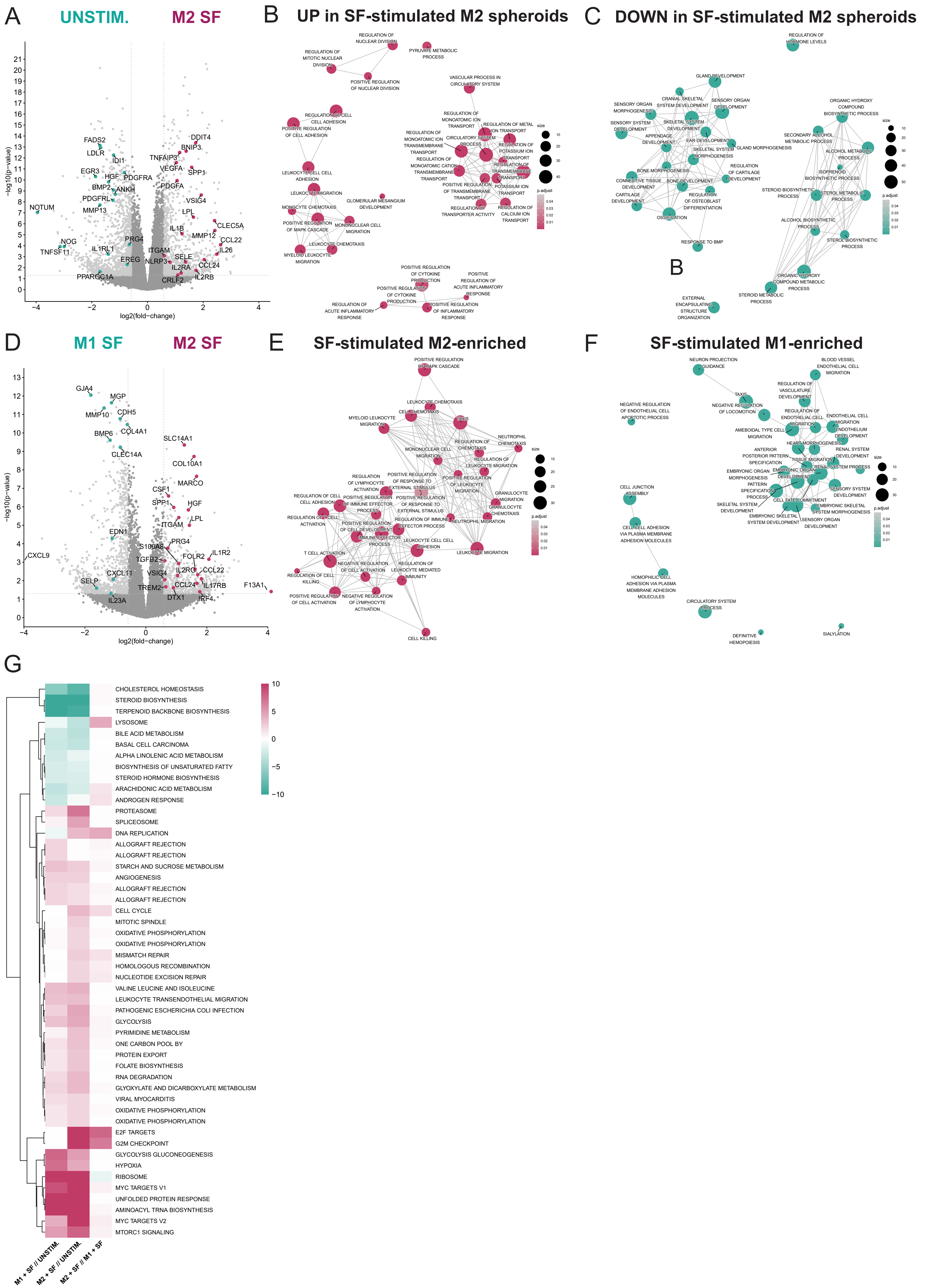
A
UNSTIM.
M2 SF
−log10(p-value)
log2(fold-change)
FADS2
LDLR
IDI1
EGR3
HGF
PDGFRA
BMP2
ANKH
PDGFRL
NOTUM
MMP13
NOG
TNFSF11
IL1RL1
PRG4
EREG
PPARGC1A
DDIT4
BNIP3
TNFAIP3
VEGFA
SPP1
PDGFA
VSIG4
LPL
CLEC5A
IL1B
MMP12
CCL22
IL26
ITGAM
SELE
NLRP3
IL2RA
CCL24
CRLF2
IL2RB
B
UP in SF-stimulated M2 spheroids
REGULATION OF NUCLEAR DIVISION
REGULATION OF MITOTIC NUCLEAR DIVISION
POSITIVE REGULATION OF NUCLEAR DIVISION
PYRUVATE METABOLIC PROCESS
VASCULAR PROCESS IN CIRCULATORY SYSTEM
REGULATION OF CELL-CELL ADHESION
POSITIVE REGULATION OF CELL ADHESION
LEUKOCYTE CELL-CELL ADHESION
LEUKOCYTE MIGRATION
MONOCYTE CHEMOTAXIS
GLOMERULAR MESANGIUM DEVELOPMENT
POSITIVE REGULATION OF MAPK CASCADE
MONONUCLEAR CELL MIGRATION
LEUKOCYTE CHEMOTAXIS
MYELOID LEUKOCYTE MIGRATION
REGULATION OF MONOATOMIC ION TRANSPORT
REGULATION OF METAL ION TRANSPORT
REGULATION OF MONOATOMIC ION TRANSMEMBRANE TRANSPORT
CIRCULATORY SYSTEM PROCESS
REGULATION OF POTASSIUM ION TRANSPORT
REGULATION OF MONOATOMIC CATION TRANSMEMBRANE TRANSPORT
REGULATION OF TRANSMEMBRANE TRANSPORT
POSITIVE REGULATION OF TRANSMEMBRANE TRANSPORT
POTASSIUM ION TRANSPORT
REGULATION OF TRANSPORTER ACTIVITY
REGULATION OF CALCIUM ION TRANSPORT
POSITIVE REGULATION OF CYTOKINE PRODUCTION
POSITIVE REGULATION OF ACUTE INFLAMMATORY RESPONSE
REGULATION OF CYTOKINE PRODUCTION
REGULATION OF ACUTE INFLAMMATORY RESPONSE
POSITIVE REGULATION OF INFLAMMATORY RESPONSE
size
p.adjust
C
DOWN in SF-stimulated M2 spheroids
REGULATION OF HORMONE LEVELS
GLAND DEVELOPMENT
CRANIAL SKELETAL SYSTEM DEVELOPMENT
SENSORY ORGAN MORPHOGENESIS
SENSORY ORGAN DEVELOPMENT
SENSORY SYSTEM DEVELOPMENT
SKELETAL SYSTEM DEVELOPMENT
APPENDAGE DEVELOPMENT
EAR DEVELOPMENT
GLAND MORPHOGENESIS
SKELETAL SYSTEM MORPHOGENESIS
BONE MORPHOGENESIS
CONNECTIVE TISSUE DEVELOPMENT
BONE DEVELOPMENT
REGULATION OF CARTILAGE DEVELOPMENT
CARTILAGE DEVELOPMENT
REGULATION OF OSTEOBLAST DIFFERENTIATION
OSSIFICATION
RESPONSE TO BMP
ORGANIC HYDROXY COMPOUND BIOSYNTHETIC PROCESS
SECONDARY ALCOHOL METABOLIC PROCESS
ALCOHOL METABOLIC PROCESS
ISOPRENOID BIOSYNTHETIC PROCESS
STEROID BIOSYNTHETIC PROCESS
STEROL METABOLIC PROCESS
ALCOHOL BIOSYNTHETIC PROCESS
STEROL BIOSYNTHETIC PROCESS
ORGANIC HYDROXY COMPOUND METABOLIC PROCESS
STEROID METABOLIC PROCESS
EXTERNAL ENCAPSULATING STRUCTURE ORGANIZATION
D
M1 SF
M2 SF
GJA4
MGP
MMP10
CDH5
BMP6
COL4A1
CLEC14A
SLC14A1
COL10A1
MARCO
CSF1
SPP1
HGF
LPL
ITGAM
EDN1
PRG4
S100A8
TGFB2
FOLR2
IL1R2
CXCL9
CXCL11
VSIG4
IL2RG
CCL22
SELP
TREM2
CCL24
IL17RB
F13A1
IL23A
DTX1
IRF4
E
SF-stimulated M2-enriched
POSITIVE REGULATION OF MAPK CASCADE
LEUKOCYTE CHEMOTAXIS
CELL CHEMOTAXIS
TAXIS
MYELOID LEUKOCYTE MIGRATION
NEUTROPHIL CHEMOTAXIS
MONONUCLEAR CELL MIGRATION
REGULATION OF CHEMOTAXIS
REGULATION OF LEUKOCYTE MIGRATION
REGULATION OF LYMPHOCYTE ACTIVATION
POSITIVE REGULATION OF RESPONSE TO EXTERNAL STIMULUS
POSITIVE REGULATION OF LEUKOCYTE MIGRATION
REGULATION OF CELL-CELL ADHESION
POSITIVE REGULATION OF IMMUNE EFFECTOR PROCESS
POSITIVE REGULATION OF RESPONSE TO EXTERNAL STIMULUS
GRANULOCYTE MIGRATION
GRANULOCYTE CHEMOTAXIS
REGULATION OF CELL-CELL ACTIVATION
POSITIVE REGULATION OF CELL DEVELOPMENT
REGULATION OF IMMUNE EFFECTOR PROCESS
NEUTROPHIL MIGRATION
IMMUNE EFFECTOR PROCESS
LEUKOCYTE CELL-CELL ADHESION
T CELL ACTIVATION
LEUKOCYTE MIGRATION
NEGATIVE REGULATION OF CELL ACTIVATION
REGULATION OF LEUKOCYTE MEDIATED IMMUNITY
REGULATION OF CELL KILLING
POSITIVE REGULATION OF CELL ACTIVATION
NEGATIVE REGULATION OF LYMPHOCYTE ACTIVATION
CELL KILLING
F
SF-stimulated M1-enriched
NEURON PROJECTION GUIDANCE
BLOOD VESSEL ENDOTHELIAL CELL MIGRATION
REGULATION OF VASCULATURE DEVELOPMENT
TAXIS
NEGATIVE REGULATION OF ENDOTHELIAL CELL APOPTOTIC PROCESS
NEGATIVE REGULATION OF LOCOMOTION
REGULATION OF ENDOTHELIAL CELL MIGRATION
ENDOTHELIAL CELL MIGRATION
AMEBOIDAL TYPE CELL MIGRATION
ENDOTHELIUM DEVELOPMENT
HEART MORPHOGENESIS
ANTERIOR POSTERIOR PATTERN SPECIFICATION
TISSUE MIGRATION
RENAL SYSTEM DEVELOPMENT
EMBRYONIC ORGAN MORPHOGENESIS
EMBRYONIC ORGAN DEVELOPMENT
RENAL SYSTEM PROCESS
PATTERN SPECIFICATION PROCESS
SENSORY SYSTEM DEVELOPMENT
CELL FATE COMMITMENT
SKELETAL SYSTEM DEVELOPMENT
EMBRYONIC SKELETAL SYSTEM MORPHOGENESIS
EMBRYONIC SKELETAL SYSTEM DEVELOPMENT
SENSORY ORGAN DEVELOPMENT
CELL JUNCTION ASSEMBLY
CELL-CELL ADHESION VIA PLASMA MEMBRANE ADHESION MOLECULES
HOMOPHILIC CELL ADHESION VIA PLASMA MEMBRANE ADHESION MOLECULES
CIRCULATORY SYSTEM PROCESS
DEFINITIVE HEMOPOIESIS
SIALYLATION
G
CHOLESTEROL HOMEOSTASIS
STEROID BIOSYNTHESIS
TERPENOID BACKBONE BIOSYNTHESIS
LYSOSOME
BILE ACID METABOLISM
BASAL CELL CARCINOMA
ALPHA LINOLENIC ACID METABOLISM
BIOSYNTHESIS OF UNSATURATED FATTY
STEROID HORMONE BIOSYNTHESIS
ARACHIDONIC ACID METABOLISM
ANDROGEN RESPONSE
PROTEASOME
SPLICEOSOME
DNA REPLICATION
ALLOGRAFT REJECTION
ALLOGRAFT REJECTION
STARCH AND SUCROSE METABOLISM
ANGIOGENESIS
ALLOGRAFT REJECTION
ALLOGRAFT REJECTION
CELL CYCLE
MITOTIC SPINDLE
OXIDATIVE PHOSPHORYLATION
OXIDATIVE PHOSPHORYLATION
MISMATCH REPAIR
HOMOLOGOUS RECOMBINATION
NUCLEOTIDE EXCISION REPAIR
VALINE LEUCINE AND ISOLEUCINE
LEUKOCYTE TRANSENDOTHELIAL MIGRATION
PATHOGENIC ESCHERICHIA COLI INFECTION
GLYCOLYSIS
PYRIMIDINE METABOLISM
ONE CARBON POOL BY
PROTEIN EXPORT
FOLATE BIOSYNTHESIS
RNA DEGRADATION
GLYOXYLATE AND DICARBOXYLATE METABOLISM
VIRAL MYOCARDITIS
OXIDATIVE PHOSPHORYLATION
OXIDATIVE PHOSPHORYLATION
E2F TARGETS
G2M CHECKPOINT
GLYCOLYSIS GLUCONEOGENESIS
HYPOXIA
RIBOSOME
MYC TARGETS V1
UNFOLDED PROTEIN RESPONSE
AMINOACYL TRNA BIOSYNTHESIS
MYC TARGETS V2
MTORC1 SIGNALING
M1 + SF // UNSTIM.
M2 + SF // UNSTIM.
M2 + SF // M1 + SF
10
5
0
−5
−10

H

Top 50 cell-type assigned genes in SF-stimulated M2 spheroids (vs UNSTIM.)

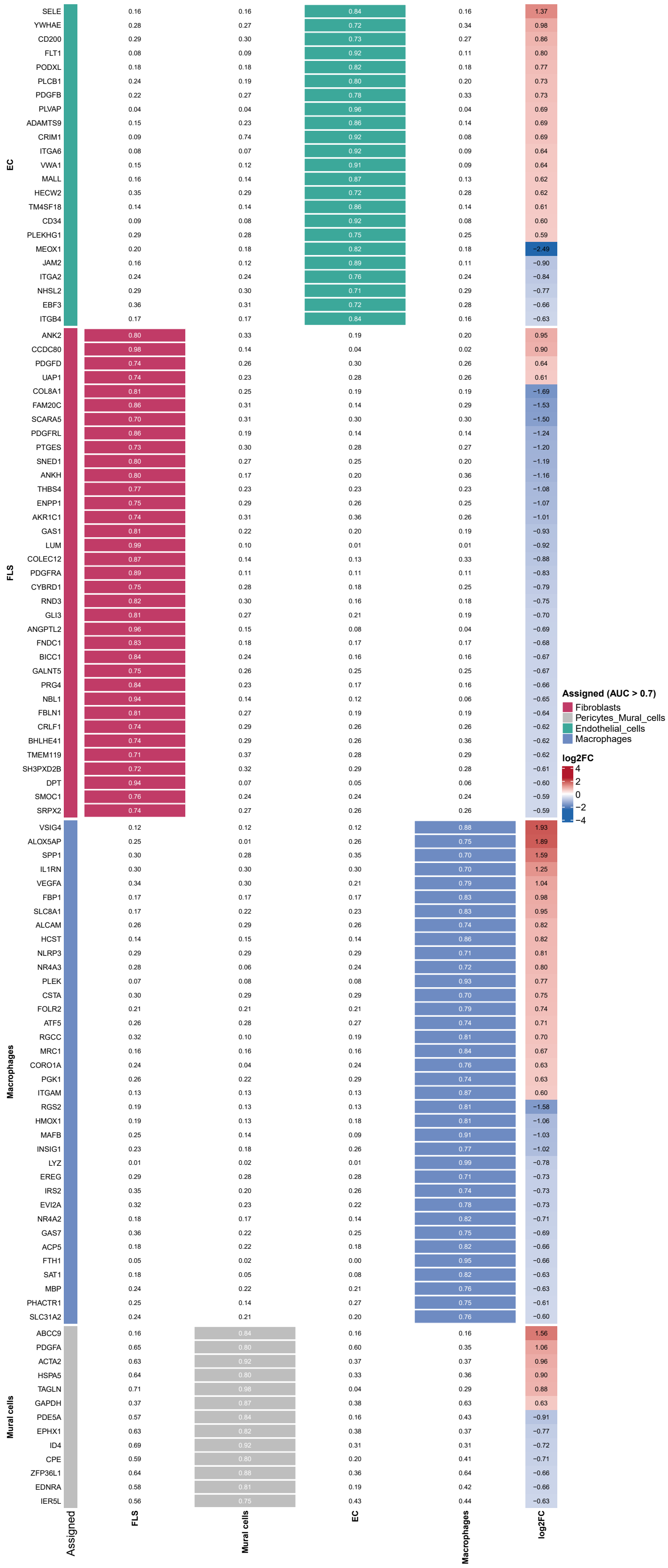

I

Top 50 cell-type assigned genes in SF-stimulated M2 spheroids (vs M1)

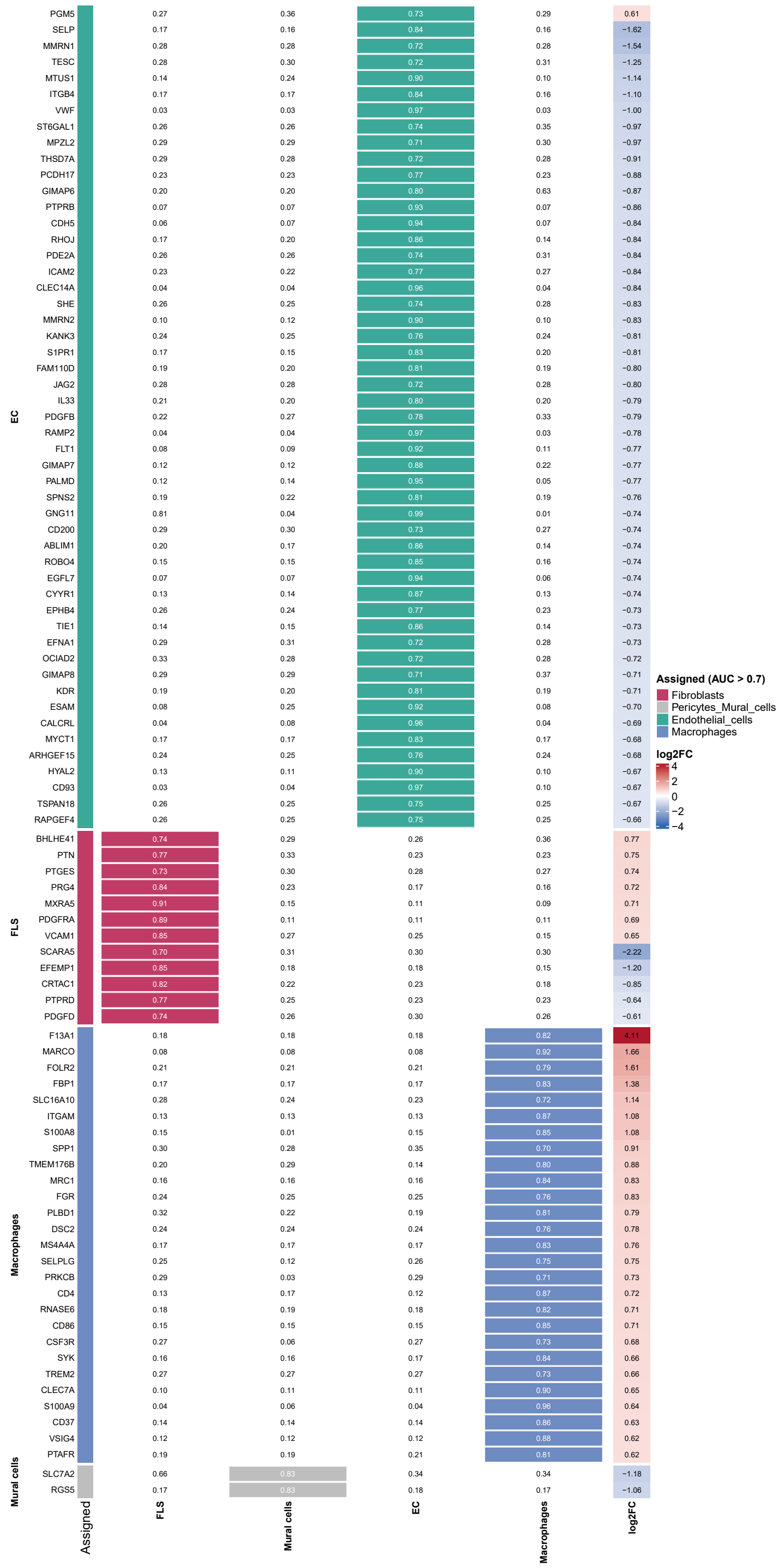

Supplement: Supplementary file 2 [file 13075_2026_3849_MOESM8_ESM.pdf]
