# Supplementary material for: Targeting inflammatory and angiogenic responses by small-molecule kinase inhibitors in a 3D macrophage-containing spheroid model of rheumatoid arthritis synovial tissue
Source: Arthritis Res Ther. 2026 Jun 23;28:163. doi: 10.1186/s13075-026-03849-z (PMC13543596; doi:10.1186/s13075-026-03849-z)
Supplement: Supplementary file 3 [file 13075_2026_3849_MOESM9_ESM.docx]

**SUPPLEMENTARY MATERIAL**

**Supplementary Material 1:** **Macrophage incorporation reshapes the transcriptional landscape toward inflammatory and myeloid-associated programs in tripartite synovial spheroids.** Differential gene expression analysis (bulk-RNA sequencing) was performed between collagen-embedded spheroids composed of RA-FLS and ECs with or without M1 macrophages after 40 h of culture in basal medium (M1-FLS-EC spheroids: n = 4; FLS-EC spheroids: n = 1). A: Volcano plot shows DEGs (p < 0.05, |FC| > 1.5) and RA-relevant genes are colored and labeled with their HGNC gene name. B: Emapplot depict Gene Ontology (GO) enrichment analysis (p.adjusted < 0.05) of the Biological Process (BP) collection performed on the upregulated DEGs (e.g. M1-FLS-EC spheroid-enriched genes). Node connection reflects shared genes between enriched GO terms and node size corresponds to the number of contributing genes. C: Bar plot of enriched KEGG and Hallmark gene sets ranked by significance (FDR < 0.05). The scale represents -log₁₀(FDR) multiplied by directionality (-1: down, +1: up) with color gradient (cyan: M1-FLS-EC spheroids, magenta: FLS-EC spheroids).

**Supplementary Material 2:** **Further characterization of the M1- and M2-spheroid models by quantitative image analysis and soluble factor measurements.** Spheroids containing M1 (cyan) or M2 macrophages (magenta), RAFLS and ECs were embedded in collagen scaffolds and fixed after 40h of culture in basal medium (n = 7-8). The area covered by the cells that migrated throughout the matrix (outgrowth) was segmented and discriminated from the core area using QuPath. A-B: Percentage of RAFLS- (A) and EC-specific (B) integrated density ($Mean Fluorescence Intensity \times Area)$calculated in the outgrowth region. C-D: IL-6 (C) and TNF (D) concentrations measured in spheroid culture supernatants by ELISA. E-J: IL-8 (E), IL-1β (F), GM-CSF (G), M-CSF (H), SPP1 (I), CXCL9 (J) were quantified by Luminex.

**Supplementary Material 3: Lists of differentially expressed genes (DEGs) identified across comparisons in the 3D spheroid model of RA synovial interactions.** Bulk RNA-sequencing was performed on collagen-embedded spheroids composed of RA-FLS and ECs alone (n = 1) or in combination with M1 or M2 macrophages (n = 4), cultured for 40 h under basal conditions (UNSTIM.) or following stimulation with RA synovial fluid (20% SF). For M1-containing spheroids, additional SF-stimulated conditions included treatment with IKKβ inhibitor (IKKβi) or tofacitinib (TOF) (n = 4). DEGs (p < 0.05, |FC| > 1.5) are provided for each comparative analysis and ranked according to fold change and directionality. Comparisons include: M1-containing versus macrophage-free spheroids under basal conditions (M1 vs No Macro UNSTIM.) and SF stimulation (M1 vs No Macro SF); M2-containing versus macrophage-free spheroids under basal conditions (M2 vs No Macro UNSTIM.) and SF stimulation (M2 vs No Macro SF); SF-stimulated versus unstimulated M1 spheroids (M1 SF vs UNSTIM.) and M2 spheroids (M2 SF vs UNSTIM.); M2-containing versus M1-containing spheroids under basal conditions (M2 vs M1 UNSTIM.) and SF stimulation (M2 vs M1 SF); and SF-stimulated M1 spheroids treated with IKKβi (M1 IKK vs SF) or tofacitinib (M1 TOF vs SF) compared with SF stimulation alone.

**Supplementary Material 4. Deconvolution of bulk RNA-seq data and cell-type attribution of differentially expressed genes in the 3D spheroid model of RA synovial interactions.** A: Estimated cellular composition of the 3D model inferred by deconvolution of bulk RNA-seq data using a published RA synovial tissue scRNA-seq reference dataset (DOI: 10.1016/j.isci.2024.109707). Relative proportions of the major cell types, e.g. fibroblasts, endothelial cells (ECs), macrophages, and mural/pericyte-like cells are shown across experimental conditions. B-E: Heatmaps displaying cell-type attribution of differentially expressed genes (DEGs) based on marker specificity derived from the reference scRNA-seq dataset. Cell-type attribution was determined using the highest AUC score across reference cell populations (AUC > 0.7). For each comparison, the top 50 DEGs ranked by log2(FC) for each assigned cell type are shown together with their corresponding AUC values. B: M2-containing versus M1-containing spheroids under basal conditions (M2 vs M1 UNSTIM.). C: SF-stimulated versus unstimulated M1-containing spheroids (M1 SF vs UNSTIM.). D: M1-containing versus macrophage-free spheroids under basal conditions (M1 vs No Macro UNSTIM.). E: M1-containing versus macrophage-free spheroids under SF stimulation (M1 vs No Macro SF).

**Supplementary Material 5:** **The growth factors VEGF/bFGF (GFs) induce spheroid outgrowth in the M1-spheroid model.** Spheroids incorporating M1 macrophages, RAFLS and ECs were embedded in collagen scaffolds and left unstimulated (UNSTIM.) or stimulated with GFs (10 ng/ml), TNF (10 ng/ml) or SF (20%) for 40h (n = 8). The area covered by the cells that migrated throughout the matrix (outgrowth) was segmented and discriminated from the core area using QuPath. A-B: Percentage of RAFLS- (A) and EC-specific (B) integrated density ($Mean Fluorescence Intensity \times Area)$quantified in the outgrowth area across GF, TNF or SF stimulation. The following parameters display GF-stimulated spheroids compared to the unstimulated conditions. C: Representative confocal pictures (10X) of the spheroids (M1 macrophages: yellow, RAFLS: magenta, ECs: cyan) after fixation. D: Outgrowth-to-core area ratio. E: Percentage of macrophage-specific integrated density quantified in the spheroid core. F: Number of macrophages counted in the surrounding matrix.

**Supplementary Material 6: NF-κB inhibition downregulates several pro-inflammatory mediators, while tofacitinib preferentially reduces IL-6 production in the TNF-stimulated M1-spheroid model.** Spheroids containing M1 macrophages, RAFLS and ECs were embedded in collagen matrices and left unstimulated (UNSTIM.), stimulated with 10 ng/ml TNF ± IKKβi (2.5 μM), tofacitinib (TOF, 5 μM) or etanercept (ETA; 100 μg/ml) for 40h (n = 8). A: IL-6 concentrations measured in spheroid culture supernatants by ELISA. B-H: IL-8 (B), IL-1β (C), SPP1 (D), GM-CSF (E), M-CSF (F), CXCL9 (G), VEGF (H) were quantified by Luminex.

**Supplementary Material 7: Small-molecule kinase inhibitors (IKKβi, tofacitinib) suppress spheroid outgrowth induced by VEGF/bFGF (GFs) stimulation, while tofacitinib and etanercept inhibit TNF-driven macrophage compaction.** Spheroids composed of M1 macrophages, RAFLS, and ECs were embedded in collagen scaffolds and cultured for 40 hours in basal medium (UNSTIM) ± GFs (10 ng/mL), TNF (10 ng/mL), or SF (20%) (n = 8). Stimulation medium was supplemented with either IKKβi (2.5 μM), tofacitinib (TOF; 5 μM) or etanercept (ETA; 100 μg/ml). The area covered by the cells that migrated throughout the matrix (outgrowth) was segmented and discriminated from the core area using QuPath. A-B: Percentage of macrophage-specific integrated density ($Mean Fluorescence Intensity \times Area)$quantified in the spheroid core (A) and number of macrophages counted in the surrounding matrix (B) upon SF stimulation ± inhibitors. C-D: Upon TNF stimulation ± inhibitors. E-G: Outgrowth-to-core area ratio (E), percentage of EC- (F) and RAFLS-specific (G) integrated densities quantified in the outgrowth area upon GF stimulation ± inhibitors.

**Supplementary Material 8: RA synovial fluid (SF) activates overlapping pathways in the M1- and M2-spheroid models while retaining subset-specific molecular features.** Differential gene expression analysis (bulk-RNA sequencing) was conducted between spheroids consisting of either M1- or M2 macrophages with RAFLS and ECs, embedded in collagen drops and cultured for 40h in basal medium (UNSTIM.) ± supplemented with SF (20%) (n = 4). A-C: SF versus UNSTIM. in M2 spheroids. D-F: SF-stimulated M2 spheroids versus M1 spheroids. Volcano plots show DEGs (p < 0.05, |FC| > 1.5) and RA-relevant genes are colored and labeled with their HGNC gene name (A, D), with CXCL9 (log2(FC)=-10.4) plotted outside the scale to preserve readability of the remaining data (D). Emapplot depict Gene Ontology (GO) enrichment analysis (p.adjusted < 0.05) of the Biological Process (BP) collection performed on the upregulated (magenta) (B, E) or the downregulated (cyan) DEGs (C, F). Node connection reflects shared genes between enriched GO terms and node size corresponds to the number of contributing genes. G: Heatmap regrouping the top 50 enriched KEGG and Hallmark gene sets ranked by significance (FDR < 0.05) for each comparative analysis: (1) SF versus UNSTIM. in M1 spheroids, (2) SF versus UNSTIM. in M2 spheroids, (3) SF-stimulated M2 versus M1 spheroids. The scale represents -log₁₀(FDR) multiplied by directionality (-1: down, +1: up) with color gradient (cyan: downregulated, magenta: upregulated). H-I: Heatmaps showing deconvolution-based cell-type attribution of DEGs using a published RA synovial tissue scRNA-seq reference dataset (DOI: 10.1016/j.isci.2024.109707). Genes were assigned to the cell population with the highest marker enrichment score (AUC > 0.7). For each comparison, the top 50 DEGs ranked by log2(FC) for each assigned cell type are shown alongside their corresponding AUC values.
